# Brexanolone, zuranolone and related neurosteroid GABA_A_ receptor positive allosteric modulators for postnatal depression

**DOI:** 10.1002/14651858.CD014624.pub2

**Published:** 2025-06-26

**Authors:** Claire A Wilson, Lindsay Robertson, Karyn Ayre, Jessica L Hendon, Sarah Dawson, Charlene Bridges, Hind Khalifeh

**Affiliations:** Institute of Psychiatry, Psychology and NeuroscienceKing's College LondonLondonUK; South London and Maudsley NHS Foundation TrustLondonUnited Kingdom; Cochrane Common Mental DisordersUniversity of YorkYorkUK; Centre for Reviews and DisseminationUniversity of YorkYorkUK; Evidence Production and MethodsCochrane Central ExecutiveLondonUK; Division of PsychiatryUniversity of EdinburghEdinburghUK; Centre for Academic Mental Health, Bristol Population Health Science InstituteUniversity of BristolBristolUK; Cochrane Central Study Identification Service, Publishing and TechnologyCochraneLondonUK

## Abstract

**Background:**

Postnatal depression – depression that occurs up to one year after a woman has given birth – is an important and common disorder that can have short‐ and long‐term adverse impacts on the mother, her child and the family as a whole. Recommended treatment for postnatal depression is psychological therapy, and for more severe depression, antidepressants. However, many antidepressants are associated with limited response. Neurosteroid gamma‐aminobutyric acid (GABA_A_) receptor positive allosteric modulators have been developed for the treatment of depression, including postnatal depression, and have a different mechanism of action than traditional antidepressants.

**Objectives:**

To assess the benefits and harms of brexanolone, zuranolone and related neurosteroid GABA_A_ receptor positive allosteric modulators compared to another active treatment (pharmacological, psychological or psychosocial), placebo or treatment as usual for postnatal depression.

**Search methods:**

We searched Cochrane Common Mental Disorders' Specialised Register, CENTRAL, MEDLINE, Embase and PsycINFO in January 2024. We also searched two international trials registries and contacted experts in the field to identify the studies that are included in the review.

**Selection criteria:**

We included randomised controlled trials (RCTs) of women with depression during the first 12 months following childbirth that compared neurosteroid GABA_A_ receptor positive allosteric modulators with any other treatment (pharmacological, psychological or psychosocial), placebo or treatment as usual.

**Data collection and analysis:**

We used standard Cochrane methodological procedures. The primary outcomes were depression response, depression remission and adverse events experienced by the mother, nursing baby, or both. The secondary outcomes were depression severity, treatment acceptability, quality of life and parenting‐ and child‐related outcomes. We grouped analyses according to whether the neurosteroid GABA_A_ receptor positive allosteric modulator was intravenous or oral. We assessed the certainty of the evidence using GRADE criteria.

**Main results:**

We identified six RCTs (674 women); all were placebo‐controlled trials. Three studies tested intravenous brexanolone; one, intravenous ganaxolone; and two studies, oral zuranolone. Sample sizes ranged from 21 to 196. All were conducted in the USA. We judged the risks of selection, performance, detection, attrition and reporting biases to mostly be low, although the risk of selection and attrition bias was unclear in two studies. The biopharmaceutical companies which made the drugs sponsored all six included studies. They appear to have had a considerable role in the design and conduct of the studies.

**Intravenous neurosteroid GABA_A_ receptor positive allosteric modulators versus placebo**

Low‐certainty evidence suggests there may be little or no difference in depression response (risk ratio (RR) 1.24, 95% confidence interval (CI) 0.74 to 2.06; I^2^ = 78%; 3 studies, 267 women) or remission (RR 1.18, 95% CI 0.59 to 2.38; I^2^ = 73%; 3 studies, 267 women) at 30 days (classified in this review as the 'early phase' of treatment: between 0 and 5 weeks from commencement of treatment). There is also probably little or no difference in the number of adverse events affecting the mother (RR 1.02, 95% CI 0.71 to 1.48; I^2^ = 46%; 4 studies, 325 women; moderate‐certainty evidence).

There is low‐certainty evidence that there may be little or no difference in depression severity (mean difference (MD) ‐4.22, 95% CI ‐8.46 to 0.02; I^2^ = 78%; 3 studies, 267 women) in the early phase (at 30 days following commencement of treatment); Hamilton Rating Scale for Depression (HAMD‐17) score range 0 to 52. Moderate‐certainty evidence suggests lower acceptability than placebo, leading to study dropout (RR 2.77, 95% CI 1.22 to 6.26; I^2^ = 0%; 3 studies, 267 women).

No studies measured quality of life or parenting‐ and child‐related outcomes.

**Oral zuranolone versus placebo**

Moderate‐certainty evidence suggests that zuranolone is probably associated with an improvement in depression response (RR 1.26, 95% CI 1.03 to 1.55; I^2^ = 13%; 2 studies, 349 women) and remission (RR 1.65, 95% CI 1.22 to 2.22; I^2^ = 0%; 2 studies, 349 women) at 45 days from commencement of treatment. Moderate‐certainty evidence also suggests that zuranolone probably increases the rate of maternal adverse events (RR 1.24, 95% CI 1.03 to 1.48; I^2^ = 0%; 2 studies, 349 women), when all adverse events are considered; the most frequent adverse event was somnolence.

Zuranolone is also probably effective in reducing depression severity at day 45 (MD ‐3.79, 95% CI ‐5.60 to ‐1.97; I^2^ = 0%; 2 studies, 349 women; moderate‐certainty evidence); HAMD‐17 score range 0 to 52. Low‐certainty evidence suggests little or no difference in terms of treatment acceptability between zuranolone and placebo (RR 0.95, 95% CI 0.50 to 1.81; I^2^ = 5%; 2 studies, 349 women).

No studies measured quality of life. One study reported the Barkin Index of Maternal Functioning (a validated measure of patient‐reported maternal functioning within the first year of childbirth), and found that zuranolone improved maternal functioning at day 45 (MD 7.20, 95% CI 1.42 to 12.98; 153 women), but the certainty of this evidence was low.

**Authors' conclusions:**

This review provides moderate‐certainty evidence that zuranolone probably improves depression response and remission but also increases maternal adverse events compared to placebo. There may be little or no difference in depression response and remission and probably little or no difference in maternal adverse events with intravenous neurosteroid GABA_A_ positive allosteric modulators such as brexanolone, compared to placebo. Evidence from this review, alongside current clinical guidelines and reference to evidence from the general adult population, could be used to inform an individualised risk‐benefit discussion with women seeking treatment for postnatal depression. However, it is difficult to make recommendations about the use of neurosteroid GABA_A_ receptor positive allosteric modulators for the treatment of postnatal depression as no studies have compared them to active treatment.

## Plain language summary

**Do brexanolone and zuranolone help women with postnatal depression?**

**Key messages**

• Compared to placebo (an inactive or 'dummy' medication), zuranolone probably helps more women by reducing their depression symptoms, but also increases the number of harmful, unwanted events they experience.

• Brexanolone may make little or no difference to women's depression symptoms compared to placebo, and probably makes little or no difference to the number of unwanted, harmful events they experience.

• We need studies that compare these medications to traditional antidepressants and talking therapies, and look at longer‐term outcomes, to better understand their benefits and harms.

**What is postnatal depression?**

Postnatal depression (also known as postpartum depression) is depression that starts within a year of a woman having a baby. Many women are affected. It can involve a persistent low mood, loss of interest or pleasure in things once enjoyed, changes in appetite and energy levels, disturbed sleep and low self‐confidence. Postnatal depression can have serious short‐ and long‐term effects on the mother, the baby and the whole family.

**How is it treated?**

There are several ways to treat postnatal depression. These include medication (such as antidepressants), talking therapies or structured support (for example, peer support). The type of treatment offered depends on the woman's choice, how severe the depression is and the presence of other illnesses. In general, women who are pregnant or breastfeeding often worry about the potential unwanted effects of medications on their baby.

Pharmaceutical companies have developed new treatments for postnatal depression that work on specific brain receptors. Brexanolone and zuranolone are two examples. As a group, they are called 'neurosteroid GABA_A_ receptor positive allosteric modulators'. Zuranolone is given as a tablet by mouth ('oral' administration); brexanolone is infused into a vein over 60 hours ('intravenous' administration). These new treatments work faster than traditional antidepressants. However, their benefits and harms are uncertain.

**What did we want to find out?**

We wanted to understand the benefits and harms of the neurosteroid GABA_A_ positive allosteric modulators, such as brexanolone and zuranolone, for treating women with postnatal depression.

**What did we do?**

In January 2024, we searched for studies of neurosteroid GABA_A_ positive allosteric modulators for women with postnatal depression. We looked for studies in which women were randomly assigned to take either the medication or a placebo. These studies give the most reliable evidence.

The outcome we focused on was how well the medication worked. This was measured by the number of women who responded well to the treatment ('response') or who no longer met criteria for depression after treatment ('remission'). We also examined whether women and/or their babies experienced unwanted, harmful (also known as 'adverse') effects from the treatment.

**What did we find?**

We found six studies involving 674 women. Three studies compared (intravenous) brexanolone with a placebo. Another study compared a different intravenous drug from the same family, called ganaxolone, with placebo. Two studies compared (oral) zuranolone with placebo. No study compared these treatments with other medications, treatment as usual (also called 'watch and wait'), talking therapies or other forms of support.

**Main results**

*Intravenous neurosteroi*d *GABA_A_ positive allosteric modulators*

• The intravenous medications (brexanolone and ganaxolone) may produce little or no improvements in depression remission, response or severity, compared to placebo.

• There is probably little or no difference in adverse events for the mothers between the intravenous medications and placebo.

• They are probably less acceptable (leading to more women leaving the studies) than placebo.

• The studies did not measure some other outcomes that we were interested in, including quality of life, parenting abilities and effect on the infant.

*Oral neurosteroi*d *GABA_A_ positive allosteric modulators*

• The oral medication, zuranolone, probably helps more women by reducing their depression symptoms (response and remission) than placebo.

• Zuranolone probably increases the number of maternal adverse events compared to placebo.

• The women may have found zuranolone and placebo to be equally acceptable (roughly the same number of women in each group left the studies early).

• Compared to placebo, zuranolone probably reduces depression severity at 5 to 12 weeks after starting the treatment.

• Zuranolone may improve the mother's parenting abilities compared to placebo.

**What are the limitations of the evidence?**

Our findings are based on only a few studies conducted up to 45 days after treatment started. Our conclusions may change if more studies are conducted. We need to better understand how these medications compare to other treatments for postnatal depression, including antidepressant medication, in the longer term and also their safety during breastfeeding.

**How current is this evidence?**

This evidence is current to January 2024.

## Summary of findings

**Summary of findings 1:** Summary of findings table ‐ Intravenous neurosteroid GABAA receptor positive allosteric modulators compared to placebo for postnatal depression

**Intravenous neurosteroid GABAA receptor positive allosteric modulators compared to placebo for postnatal depression**						
**Patient or population:** postnatal depression **Setting:** outpatients **Intervention:** intravenous neurosteroid GABAA receptor positive allosteric modulators **Comparison:** placebo						
**Outcomes**	Anticipated absolute effects^*^ (95% CI)		**Relative effect (95% CI)**	**№ of participants (studies)**	**Certainty of the evidence (GRADE)**	**Comments**
	**Risk with placebo**	**Risk with intravenous neurosteroid GABAA receptor positive allosteric modulators**				
Depression response, defined as number of participants with ≥ 50% reduction in Hamilton Rating Scale for Depression (HAMD‐17) total score in the early phase (0 to 5 weeks) 30 days post infusion	694 per 1000	**860 per 1000** (513 to 1000)	**RR 1.24** (0.74 to 2.06)	267 (3 RCTs)	⊕⊕⊝⊝ Low^a^	Intravenous neurosteroid GABAA positive allosteric modulators may result in little or no difference in depression response.
Depression remission, defined as number of participants with HAMD‐17 total score ≤ 7 in the early phase (0 to 5 weeks) 30 days post infusion	477 per 1000	**563 per 1000** (282 to 1000)	**RR 1.18** (0.59 to 2.38)	267 (3 RCTs)	⊕⊕⊝⊝ Low^a^	Intravenous neurosteroid GABAA positive allosteric modulators may result in little to no difference in depression remission.
Any adverse events (mother)	439 per 1000	**448 per 1000** (312 to 649)	**RR 1.02** (0.71 to 1.48)	325 (4 RCTs)	⊕⊕⊕⊝ Moderate^b^	Intravenous neurosteroid GABAA positive allosteric modulators probably result in little to no difference in adverse events.
Depression severity, defined as mean change from baseline in HAMD‐17 total score in the early phase (0 to 5 weeks) 30 days post infusion	The mean depression severity, defined as mean change from baseline in HAMD‐17 total score in the early phase (0 to 5 weeks) 30 days post infusion was **0**	MD **4.22 lower** (8.46 lower to 0.02 higher)	‐	267 (3 RCTs)	⊕⊕⊝⊝ Low^c^	Intravenous neurosteroid GABAA positive allosteric modulators may result in little to no difference in severity of depression in the early phase.
Treatment acceptability, measured by number of dropouts	54 per 1000	**150 per 1000** (66 to 338)	**RR 2.77** (1.22 to 6.26)	267 (3 RCTs)	⊕⊕⊕⊝ Moderate^d^	Intravenous neurosteroid GABAA positive allosteric modulators are probably less acceptable, leading to dropout.
Quality of life	0 per 1000	**0 per 1000** (0 to 0)	Not estimable	(0 RCTs)	‐	No study measured this outcome.
Parenting‐related and child‐related outcomes	0 per 1000	**0 per 1000** (0 to 0)	Not estimable	(0 RCTs)	‐	No study measured this outcome.
*The risk in the intervention group (and its 95% confidence interval) is based on the assumed risk in the comparison group and the **relative effect** of the intervention (and its 95% CI). **CI:** confidence interval; **MD:** mean difference; **RR:** risk ratio						
**GRADE Working Group grades of evidence** **High certainty:** we are very confident that the true effect lies close to that of the estimate of the effect. **Moderate certainty:** we are moderately confident in the effect estimate: the true effect is likely to be close to the estimate of the effect, but there is a possibility that it is substantially different. **Low certainty:** our confidence in the effect estimate is limited: the true effect may be substantially different from the estimate of the effect. **Very low certainty:** we have very little confidence in the effect estimate: the true effect is likely to be substantially different from the estimate of effect.						
See interactive version of this table: https://gdt.gradepro.org/presentations/#/isof/isof_question_revman_web_450958831006341011.						

^a^ Downgraded one level for inconsistency as effect estimates vary between trials and there is substantial heterogeneity (I2 = 71%). Downgraded one level for imprecision as the sample size may not be large enough to detect a precise effect estimate, and the confidence intervals are wide and include an appreciable benefit and an appreciable harm. ^b^ Downgraded one level for imprecision as the sample size may not be large enough to detect a precise effect estimate, and the confidence intervals are wide and include an appreciable benefit and an appreciable harm. ^c^ Downgraded one level for inconsistency as I2 represents substantial heterogeneity. Downgraded one level for imprecision as the sample size may not be large enough to detect a precise effect estimate, and the confidence intervals are wide and include an appreciable benefit and an appreciable harm. ^d^ Downgraded one level for imprecision as the sample size may not be large enough to detect a precise effect estimate, and the confidence intervals are wide.

**Summary of findings 2:** Summary of findings table ‐ Zuranolone compared to placebo for postnatal depression

**Zuranolone compared to placebo for postnatal depression**						
**Patient or population:** postnatal depression **Setting:** outpatients **Intervention:** zuranolone **Comparison:** placebo						
**Outcomes**	Anticipated absolute effects^*^ (95% CI)		**Relative effect (95% CI)**	**№ of participants (studies)**	**Certainty of the evidence (GRADE)**	**Comments**
	**Risk with placebo**	**Risk with zuranolone**				
Depression response, defined as number of participants with ≥ 50% reduction in Hamilton Rating Scale for Depression (HAMD‐17) total score in the acute phase (5 to 12 weeks)	489 per 1000	**616 per 1000** (503 to 757)	**RR 1.26** (1.03 to 1.55)	349 (2 RCTs)	⊕⊕⊕⊝ Moderate^a^	Zuranolone is probably associated with an improvement in depression response.
Depression remission, defined as number of participants with HAMD‐17 total score ≤ 7 in the acute phase (5 to 12 weeks)	264 per 1000	**436 per 1000** (323 to 587)	**RR 1.65** (1.22 to 2.22)	349 (2 RCTs)	⊕⊕⊕⊝ Moderate^a^	Zuranolone is probably associated with an improvement in depression remission.
Any adverse events (mother)	517 per 1000	**641 per 1000** (533 to 766)	**RR 1.24** (1.03 to 1.48)	349 (2 RCTs)	⊕⊕⊕⊝ Moderate^a^	Zuranolone probably increases the rate of maternal adverse events.
Depression severity, defined as mean change from baseline in HAMD‐17 total score in the acute phase (5 to 12 weeks)	The mean depression severity, defined as mean change from baseline in HAMD‐17 total score in the acute phase (5 to 12 weeks) was **0**	MD **3.79 lower** (5.6 lower to 1.97 lower)	‐	349 (2 RCTs)	⊕⊕⊕⊝ Moderate^a^	Zuranolone is probably effective in reducing depression severity in the acute phase.
Treatment acceptability, measured by number of dropouts	109 per 1000	**104 per 1000** (55 to 198)	**RR 0.95** (0.50 to 1.81)	349 (2 RCTs)	⊕⊕⊝⊝ Low^b^	The evidence suggests little or no difference in treatment acceptability.
Quality of life	0 per 1000	**0 per 1000** (0 to 0)	Not estimable	(0 RCTs)	‐	No study measured this outcome.
Parenting‐related outcomes in the acute phase (5 to 12 weeks) assessed with: Barkin Index of Maternal Functioning (higher score indicates better functioning)	The mean parenting‐related outcomes in the acute phase (5 to 12 weeks) was **0**	MD **7.2 higher** (1.42 higher to 12.98 higher)	‐	153 (1 RCT)	⊕⊕⊝⊝ Low^c^	Zuranolone may improve maternal functioning.
*The risk in the intervention group (and its 95% confidence interval) is based on the assumed risk in the comparison group and the **relative effect** of the intervention (and its 95% CI). **CI:** confidence interval; **MD:** mean difference; **RR:** risk ratio						
**GRADE Working Group grades of evidence** **High certainty:** we are very confident that the true effect lies close to that of the estimate of the effect. **Moderate certainty:** we are moderately confident in the effect estimate: the true effect is likely to be close to the estimate of the effect, but there is a possibility that it is substantially different. **Low certainty:** our confidence in the effect estimate is limited: the true effect may be substantially different from the estimate of the effect. **Very low certainty:** we have very little confidence in the effect estimate: the true effect is likely to be substantially different from the estimate of effect.						
See interactive version of this table: https://gdt.gradepro.org/presentations/#/isof/isof_question_revman_web_450389414078010547.						

^a^ Downgraded one level for imprecision as the sample size may not be large enough to calculate a precise effect estimate. ^b^ Downgraded two levels for imprecision as the sample size may not be large enough to calculate a precise effect estimate, and the confidence intervals are wide and include an appreciable benefit and an appreciable harm. ^c^ Downgraded two levels for imprecision as the sample size may not be large enough to calculate a precise effect estimate, and the confidence intervals are wide.

## Background

### Description of the condition

Postnatal depression (PND) – depression that occurs up to one year after a woman has given birth – is an important and common disorder that can have short‐ and long‐term adverse impacts on the mother, her child and the family as a whole (Howard 2014; Stein 2014). Perinatal suicide, which is closely linked to PND, is an important contributor to maternal mortality (Grigoriadis 2017; Khalifeh 2016; Knight 2019). PND is associated with impaired maternal‐infant attachment and internalising and externalising problems in children of mothers who have PND, particularly where depression is severe and persistent and there are familial comorbidities (Stein 2014). PND has a similar clinical presentation to depression in the general population (Howard 2014; Stewart 2019). It is characterised by persistent low mood and loss of pleasure or interests, occurring with associated symptoms such as changes in appetite and energy levels, disturbed sleep and low self‐confidence (Howard 2014; WHO 2018). The 11^th^ revision of the *International Classification of Diseases* (ICD‐11) and the fifth revision of the *Diagnostic and Statistical Manual of Mental Disorders* (DSM‐5) recommend the use of generic (non‐perinatal) mood disorder diagnostic categories for depression occurring in the postnatal period, in recognition of the absence for clear evidence of a distinct postnatal depressive clinical syndrome (APA 2013; O'Hara 2013; WHO 2018). However, they allow for the use of a secondary perinatal diagnostic category (in ICD‐11) or specifier (in DSM‐5) for depression occurring in pregnancy or within four to six weeks of childbirth.

It is important to distinguish PND from less severe, short‐lived conditions, such as the 'baby blues', which occurs in around 50% of women and resolves spontaneously within a few weeks (Howard 2014; Stewart 2019). On the other end of the severity spectrum, it is important to recognise the severe psychiatric emergency of postpartum psychosis: a rare condition affecting one to two women per 1000 in the general population, where admission is recommended to mitigate risks to mother and baby (Jones 2014). Clinically, PND is often comorbid with other conditions, particularly anxiety disorders (Stewart 2019).

In the UK and internationally, research and clinical practice have most commonly defined PND as that occurring within one year of childbirth (Howard 2014; NICE 2020; Stewart 2016; Stewart 2019). We use this definition in this review. However, there is no clear consensus on a definitive timeframe. Past research, practice guidelines and diagnostic classifications have variably defined PND as depression occurring within four weeks to 12 months of delivery (O'Hara 2013; Stewart 2019). In the absence of a consensus, it has been helpfully proposed that the relevant timeframe is likely to vary according to study aim, with shorter timeframes being most relevant for biological studies and longer timeframes for prevention or treatment studies (O'Hara 2013).

A recent systematic review of the prevalence and incidence of perinatal (i.e. antenatal and postnatal) depression estimated a pooled prevalence for PND of 9.5% (95% confidence interval (CI) 8.90 to 10.10) in high‐income settings and 18.7% (95% CI 17.80 to 19.70) in low‐ and middle‐income settings (Woody 2017). No significant differences were found between studies using different diagnostic tools (for example, a standardised structured diagnostic interview based on DSM criteria versus those using symptom scales (such as the Edinburgh Postnatal Depression Scale (EPDS)). There are few incidence studies (Woody 2017), and contradictory evidence on whether depression is more likely to occur in the postnatal period than at other times in a woman’s life (Munk‐Olsen 2006; Silverman 2019; Stewart 2019), with some evidence that the risk is elevated specifically for more severe illness requiring admission (Munk‐Olsen 2009; Munk‐Olsen 2016). Amongst women who experience PND, around a third have also had antenatal depression and a third have had depression prior to conceiving (Wisner 2013).

Most women with PND recover within a few months but about 30% of episodes last beyond the first postpartum year (Goodman 2004). Women who have had PND also have a high risk (about 40%) of both postnatal and non‐postnatal relapse (Cooper 1995; Wisner 2004).

### Description of the intervention

British perinatal guidelines recommend a stepped‐care approach to treating PND, with antidepressants advised for women experiencing more severe symptoms, either alone or in combination with psychological therapy (McAllister‐Williams 2017; NICE 2020). Selective serotonin reuptake inhibitors (SSRIs) have been the most commonly prescribed antidepressants during pregnancy and the postpartum period, and have a relatively favourable reproductive safety profile (McAllister‐Williams 2017).

However, many antidepressants are associated with limited response, or extended time to response, remission, or both (Brown 2021). These antidepressants do not directly relate to the putative pathophysiology of PND. GABA (gamma‐aminobutyric acid) is an inhibitory neurotransmitter in the central nervous system. Pre‐clinical and clinical studies in PND have highlighted the potential role of dysfunctional GABAergic signalling, suggesting that positive allosteric modulation of GABA_A_ receptors may provide a promising mechanism of action for emerging pharmacotherapy (Meltzer‐Brody 2020). Such insights into the role of GABAergic signalling in PND have led to the development of a number of PND treatments that act as allosteric modulators of GABA_A_ receptors. These include an intravenous (IV) infusion of a neuroactive steroid, allopregnanolone, known as brexanolone (also known as Zulresso or SAGE‐547). In 2019, the United States' Food and Drug Administration (FDA) approved the use of brexanolone for the treatment of PND in women, making it the first medication approved specifically for the treatment of PND. However, it is not yet approved for use in the UK. Brexanolone is administered intravenously over 60 hours with close monitoring, due to concerns about the risk of excessive sedation. Since brexanolone, other inhibitory neurosteroids have also been developed. In 2023, the FDA approved another version of allopregnanolone modified for oral administration: zuranolone (also known as SAGE‐217). It is administered as a 14‐day oral course. As with brexanolone, it has yet to be approved for use in the UK.

The safety of medication for PND while breastfeeding is also an important consideration for any PND treatment. PND has potential adverse effects for mother and baby (Howard 2014; Stein 2014). These adverse effects need to be weighed against the risks of medication exposure via breast milk, which are sometimes uncertain (McAllister‐Williams 2017).

### How the intervention might work

While there are some possible similarities in the pathophysiology of PND and depression occurring outside the perinatal period, such as dysregulation of the hypothalamic‐pituitary (HPA) axis (Maguire 2019), there are also physiological changes unique to pregnancy and evidence to support a unique pathophysiology of PND (Meltzer‐Brody 2020). A number of neuroendocrine changes have been observed in PND, including changes in GABAergic signalling. In human and animal models of PND, alterations in levels of allosteric modulators of GABA_A_ have been noted across the perinatal period (Meltzer‐Brody 2020). One such GABA_A_ receptor modulator is allopregnanolone, which is a metabolite of progesterone. Allopregnanolone levels mirror those of progesterone in the perinatal period, in that they rise during pregnancy and fall after childbirth (Luisi 2000; Paoletti 2006). Women up to six months postpartum have been observed to have lower levels of allopregnanolone than non‐pregnant women, although not all studies have found a difference in allopregnanolone levels between depressed and non‐depressed postnatal women (Epperson 2006; Maguire 2019). However, postpartum allopregnanolone levels have been observed to be positively correlated with altered functional connectivity in the brains of women with PND, further supporting a relationship between allopregnanolone levels and PND (Deligiannidis 2019). Brexanolone is an intravenous formulation and zuranolone an oral formulation of allopregnanolone, and there are other synthetic analogues of allopregnanolone under development, which serve as positive allosteric modulators of the GABA_A_ receptor. These include ganaxolone (also known as CCD‐1042), which can be administered both orally and intravenously.

### Why it is important to do this review

PND is a common problem that can have adverse short‐ and long‐term effects on the mother, her child and the wider family, including problems with mother‐infant attachment, emotional and behavioural problems in children and, rarely, maternal suicide (Howard 2014; Khalifeh 2016; Stein 2014). There is an urgent need for updated high‐quality evidence to inform treatment for the growing number of women accessing help for PND.

Many women who are pregnant or postnatal have a preference for psychological therapy over medication, and may be anxious about the potential adverse effects of medication use on the unborn or breastfeeding baby (O'Mahen 2008). However, antidepressants are recommended for treating severe PND and moderate PND that has not responded to psychological therapy, and for preventing relapse amongst women with a history of severe depressive illness (NICE 2020). Nevertheless, some women may not respond to antidepressant medication, necessitating the development of alternative pharmacological interventions. From the current understanding of PND's pathophysiology, brexanolone, zuranolone and related neurosteroid GABA_A_ receptor positive allosteric modulators have been developed as promising new treatments for PND. However, their benefits and harms have not yet been reviewed.

## Objectives

To assess the benefits and harms of brexanolone, zuranolone and related neurosteroid GABA_A_ receptor positive allosteric modulators compared to another active treatment (pharmacological, psychological or psychosocial), placebo or treatment as usual for PND.

## Methods

### Criteria for considering studies for this review

#### Types of studies

We included all published and unpublished randomised controlled trials (RCTs) and cluster‐RCTs. We planned to include trials employing a cross‐over design, but excluded all other study designs, including non‐randomised studies.

#### Types of participants

##### Participant characteristics

We included women of any age with PND. The eligible period of treatment onset was from delivery to 12 months postnatal. For our definition of the term 'woman', see Appendix 1.

##### Diagnosis

We used a broad definition of PND to include all women depressed during the first 12 months postnatal, regardless of time of onset of depression (i.e. including women whose depression started during or before pregnancy). We included trials in which women met criteria for depression, diagnosed using any of the following: a validated screening measure; a standard observer‐rated diagnostic instrument from a recognised diagnostic scheme (for example, DSM‐5 (APA 2013) or ICD‐11 (WHO 2018)); or by other standardised criteria (for example, the Research Diagnostic Criteria (RDC) (Spitzer 1978)). The threshold scores we used for the respective scales were those adopted by the trial investigators.

##### Comorbidities

We included studies that enrolled women with comorbid physical conditions or psychological disorders (for example, anxiety), provided the comorbidity was not the focus of the study.

##### Setting

We did not impose any restrictions on the type of study setting.

#### Types of interventions

##### Experimental interventions

We included brexanolone (also known as Zulresso or SAGE‐547), zuranolone (also known as SAGE‐217) and related neurosteroid GABA_A_ receptor positive allosteric modulators. Brexanolone is an exogenous version of the inhibitory neurosteroid allopregnanolone. Zuranolone is an inhibitory neurosteroid that is structurally similar to allopregnanolone, as are other related neurosteroid GABA_A_ receptor positive allosteric modulators, including but not limited to ganaxolone (also known as CCD‐1042). Interventions could be given at any dose, alone or in combination with another treatment, initiated in at least one trial arm.

##### Comparator interventions

- Placebo
- Other pharmacological interventions (for example, antidepressants)
- Any other treatment, including:
  - treatment as usual (including, but not limited to, ‘watch and wait’, regular visits with a care co‐ordinator or interventions aimed at addressing social risk factors)​​;
  - psychological interventions (for example, cognitive behavioural therapy (CBT) or interpersonal therapy);
  - psychosocial interventions (for example, peer support or non‐directive counselling).

#### Types of outcome measures

We included studies that met the above inclusion criteria regardless of whether they reported the following outcomes.

##### Primary outcomes

- Depression response, using dichotomous response measures as reported in the individual studies and defined by the study authors. Response is typically measured as the number of participants with at least a 50% reduction in the total score on a standardised depression scale.
- Depression remission, using dichotomous response measures as reported in the individual studies and defined by the study authors. Remission is typically measured as the number of participants whose scores fall below a predefined threshold on a standardised depression scale.
- Adverse events (or side effects) experienced by:
  - mother;
  - nursing baby.

##### Secondary outcomes

- Depression severity (continuous data), assessed using self‐reported rating scales, such as the Edinburgh Postnatal Depression Scale (EPDS, Cox 1987) or clinician‐rated scales, such as the Hamilton Rating Scale for Depression (HAMD, Hamilton 1967)
- Treatment acceptability, assessed directly by questioning trial participants and indirectly by dropout rates
- Quality of life (for example, measured using the 36‐item Short Form health survey (SF‐36, Ware 1992))
- Parenting‐related and child‐related outcomes (for example, maternal relationship with the baby and the establishment or continuation of breastfeeding)

##### Timing of outcome assessment

- Early phase: between 0 and 5 weeks from commencement of treatment
- Acute phase: between 5 and 12 weeks from commencement of treatment
- Continuation phase: more than 12 weeks from commencement of treatment

Our key outcome was the acute phase treatment response (between 5 and 12 weeks). We believe this outcome is the most clinically meaningful; it is also the key outcome analysed in our review of traditional antidepressant treatment for PND (Brown 2021). Where this was reported, we used any additional early and continuation phase responses as secondary outcomes.

### Search methods for identification of studies

We identified all studies that might describe brexanolone (Zulresso or SAGE‐547), zuranolone (SAGE‐217) and any other related neurosteroid GABA_A_ receptor positive allosteric modulators for the treatment of PND.

#### Electronic searches

Cochrane Information Specialists (SD and CB) searched the following biomedical databases using relevant keywords, subject headings (controlled vocabularies) and search syntax appropriate to each resource (Appendix 2).

- Cochrane Central Register of Controlled Trials (CENTRAL; 2024, Issue 3), in the Cochrane Library
- MEDLINE Ovid (1946 to 24 January 2024)
- Embase Ovid (1980 to 24 January 2024)
- PsycINFO Ovid (inception to 24 January 2024)

We also searched two international trial registers (ClinicalTrials.gov and the World Health Organization (WHO) International Clinical Trials Registry Platform (ICTRP)) on 24 January 2024, using drug terms only.

Although brexanolone only received regulatory approval from the FDA in March 2019 and zuranolone in August 2023, we did not apply any date restrictions to the search to ensure we captured all earlier (pre‐regulatory) studies. We did not apply any restrictions on language, publication status or study design in the searches.

#### Searching other resources

##### Regulatory documents

We searched for relevant regulatory approval documents (reviews) submitted by Sage Therapeutics Inc. to the US Food and Drug Administration, by searching Drugs@FDA: FDA-Approved Drugs for Zulresso (NDA 211371).

##### Reference lists

We performed forward and backward citation tracking of all included studies to identify additional studies missed from the original electronic searches (for example, unpublished or in‐press citations).

##### Personal communication

We requested additional data where necessary, or information on ongoing or completed but unpublished trials from the following sources.

- Sage Therapeutics Inc. (developers of brexanolone (Zulresso) and zuranolone (SAGE‐217))
- Marinus Pharmaceuticals (developers of ganaxolone (CCD‐1042))
- Any other pharmaceutical company or research institute involved in any of the included trials (as funder, sponsor or trialist)
- Authors of included trials published within the last five years
- The International Marcé Society for Perinatal Mental Health

### Data collection and analysis

#### Selection of studies

We managed records retrieved by the literature search in Covidence (Covidence). Two review authors (CAW, LR, KA or JLH) independently inspected abstracts retrieved from the search. We obtained the full‐text articles of any potentially relevant publications. Two review authors (CAW, LR, KA or JLH) independently assessed the full‐text articles for inclusion based on the defined inclusion criteria. We resolved any disagreements through discussion or by recourse to another review author (HK).

We recorded reasons for excluding ineligible studies. We collated multiple reports that related to the same study so that each study, rather than each report, was the unit of interest in the review. We recorded the study selection process in a PRISMA flowchart (see Figure 1), and we reported details of all included studies (see Characteristics of included studies).

**1:**
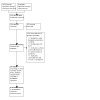
PRISMA flow diagram

#### Data extraction and management

Using Covidence, we extracted the following data from the included studies.

- **Methods:** date of study, study design, study setting, details of blinding/allocation concealment, total duration of study, details of any 'run‐in' period, number of study centres and location, and withdrawals.
- **Participants:** total number and number in each group, inclusion and exclusion criteria, mean age, age range, severity and duration of condition, diagnostic criteria, time elapsed between delivery of baby and commencement of treatment, time of onset of current depressive symptoms, physical and mental health comorbidities.
- **Interventions:** number of intervention groups, type of interventions and comparisons, duration of intervention and key details (for example, dosage, adherence, quality of delivery), concomitant medications and excluded medications.
- **Outcomes:** details of measures used to assess outcomes (for example, details of validation), primary and secondary outcomes specified and collected, time points reported and adverse events.
- **Analysis:** statistical techniques used, unit of analysis for each outcome, subgroup analyses and number of participants followed up in each study group.
- **Notes:** publication type, funding for trial and notable conflicts of interest of trial authors.

Two review authors (CAW, LR or JLH) independently extracted data from included studies. We resolved any disagreements through discussion or by recourse to another review author (HK).

We imported data into Review Manager (RevMan) for analysis (RevMan 2024).

##### Main comparisons

The main planned comparisons were as follows.

- Intravenous neurosteroid GABA_A_ receptor positive allosteric modulators versus placebo
- Intravenous neurosteroid GABA_A_ receptor positive allosteric modulators versus another pharmacological intervention
- Intravenous neurosteroid GABA_A_ receptor positive allosteric modulators versus any other intervention (for example, treatment as usual, psychological or psychosocial intervention)
- Oral neurosteroid GABA_A_ receptor positive allosteric modulators versus placebo
- Oral neurosteroid GABA_A_ receptor positive allosteric modulators versus another pharmacological intervention
- Oral neurosteroid GABA_A_ receptor positive allosteric modulators versus any other intervention (for example, treatment as usual, psychological or psychosocial intervention)

#### Assessment of risk of bias in included studies

Two review authors (CAW, LR or JLH) independently assessed the risk of bias for each study using the criteria outlined in the *Cochrane Handbook for Systematic Reviews of Interventions* (Higgins 2011). We resolved any disagreements through discussion or by recourse to another review author (HK). Cochrane's original risk of bias tool assesses bias according to the following seven domains:

- random sequence generation;
- allocation concealment;
- blinding of participants and personnel;
- blinding of outcome assessment;
- incomplete outcome data;
- selective outcome reporting;
- other bias (adherence to medication), funding source, conflicts of interest.

We used RevMan 2024 to produce risk of bias figures based on our assessment of each domain as low, high or unclear risk. We minimised the use of the 'unclear' category by contacting trial authors for further information as needed.

#### Measures of treatment effect

##### Dichotomous data

We used the risk ratio (RR) and its 95% confidence interval (CI) for dichotomous data (Bland 2000).

##### Continuous data

As all the included studies used the same outcome measure for comparison, we used mean differences (or least squares mean difference) and their standard error (SE) in meta‐analyses using the generic inverse‐variance method.

If studies had reported a combination of change‐from‐baseline and endpoint data, we planned to convert data onto the same scale (i.e. change‐from‐baseline or endpoint). We anticipated this would require estimating or imputing the endpoint or change‐from‐baseline standard deviation (SD), for which we planned to use methods described in the *Cochrane Handbook for Systematic Reviews of Interventions* (Higgins 2023).

Deligiannidis 2023 reported the confidence intervals for the least squares mean difference. We calculated the standard error using the equation in section 6.5.2.3 of the *Cochrane Handbook* (Higgins 2023).

#### Unit of analysis issues

Trials with more than two arms can complicate pairwise meta‐analysis. In line with guidance in the *Cochrane Handbook* (Higgins 2023), we employed the following approach for studies with two or more active treatment arms. For dichotomous outcomes, we combined active treatment groups into a single arm for comparison with the control group, combining event counts and sample sizes using the formula set out in Table 6.5.a in Chapter 6 of the *Cochrane Handbook* (Higgins 2023).

For continuous outcomes, we pooled the mean differences and SEs. For NCT03228394, we combined the ganaxolone groups and the placebo groups using the formula set out in Table 6.5.a in Chapter 6 of the *Cochrane Handbook.* However, we could not combine the two brexanolone groups in Meltzer‐Brody 2018 Study 1: the authors reported the mean differences relative to the placebo group, and the equation in Table 6.5.a can only be used for independent groups. Instead, we treated the two groups as two separate studies. Although this could introduce a unit of analysis issue, we felt it was unlikely to affect the overall result.

#### Dealing with missing data

At some degree of loss to follow‐up, data lose credibility (Xia 2009). However, due to the small evidence base, we included studies with greater than 50% dropout. We planned to assess the impact of data lost to follow‐up in sensitivity analyses; however, there were insufficient data to conduct these analyses.

Where included trials presented binary outcome data for women who were lost to follow‐up, we reported the data. We presented data on a 'once randomised, always analyse' basis, assuming an intention‐to‐treat (ITT) analysis. We assumed that women lost to follow‐up had a negative outcome, except for the outcome of death. For example, for the outcome of depression remission, we assumed that none of the women lost to follow‐up experienced depression remission.

Where specific data were not reported but appeared to have been collected, we contacted the relevant study authors, pharmaceutical company or both, to request this data.

#### Assessment of heterogeneity

Where we had sufficient data for a meta‐analysis, we assessed statistical heterogeneity visually by studying the degree of overlap of the CIs for individual studies in a forest plot. We also carried out more formal assessments using the I^2^ statistic. The I^2^ statistic provides only an approximate estimate of the variability due to heterogeneity, so we used the following overlapping bands to guide our interpretation of the I^2^ statistic, as suggested in the *Cochrane Handbook* (Deeks 2023):

- 0% to 40% might not be important heterogeneity;
- 30% to 60% may represent moderate heterogeneity;
- 50% to 90% may represent substantial heterogeneity;
- 75% to 100% represents considerable heterogeneity.

#### Assessment of reporting biases

We planned to generate funnel plots and inspect them visually for asymmetry. However, none of our meta‐analyses included at least 10 studies with data for the primary outcomes.

#### Data synthesis

Where data permitted, we conducted a random‐effects meta‐analysis to synthesise primary outcome data on depression response, remission and adverse events for our six pre‐specified comparisons.

In our protocol (Wilson 2021), we specified that three or more studies would be required for meta‐analysis. However, after consulting with the Cochrane Editorial team, we decided to meta‐analyse the zuranolone studies (of which there were only two), based on the clinical homogeneity of these studies.

We used RevMan for meta‐analysis (RevMan 2024).

We narratively summarised results when there were insufficient data to permit meta‐analysis.

#### Subgroup analysis and investigation of heterogeneity

We had planned to perform a number of subgroup analyses, as outlined in our protocol (Wilson 2021). However, there were sufficient data to conduct only one planned subgroup analysis; namely, isolating for an individual drug or compound (in this case, brexanolone).

We explored and commented on any observed clinical heterogeneity – for example, due to different definitions of PND or the use of different diagnostic tools – in the Discussion.

#### Sensitivity analysis

We planned to conduct a range of sensitivity analyses to explore the robustness of pooled estimates to decisions made in the conduct of the systematic review, as outlined in our protocol (Wilson 2021). Insufficient data were available to permit any of these sensitivity analyses.

#### Summary of findings and assessment of the certainty of the evidence

We created summary of findings tables for two comparisons: intravenous neurosteroid GABA_A_ positive allosteric modulators versus placebo (Table 1) and zuranolone (oral neurosteroid GABA_A_ positive allosteric modulator) versus placebo (Table 2). We included all pre‐specified primary and secondary outcomes. Where possible, we presented data for the acute phase treatment response (between 5 and 12 weeks) as our primary outcome in these summary of findings tables.

We used the five GRADE considerations (study limitations, consistency of effect, imprecision, indirectness and publication bias) to assess the certainty of the body of evidence as it relates to the studies that contributed data to the meta‐analyses for the pre‐specified outcomes. We used methods and recommendations described in Chapter 14 of the *Cochrane Handbook* (Schünemann 2019), using GRADEpro software (GRADEpro GDT 2015). We justified all decisions to downgrade the certainty of the evidence using footnotes, and we made comments to aid the reader's understanding of the review where necessary.

Two review authors (CAW, LR) independently assessed the certainty of the evidence and resolved disagreements through discussion or by consulting a third review author (HK). Judgements were justified, documented and incorporated into the reporting of results for each outcome.

##### Reaching conclusions

We based our conclusions only on findings from the quantitative or narrative synthesis of included studies for this review. We avoided making definitive recommendations for clinical practice, and our implications for research suggest priorities for future research and outline the remaining uncertainties in the research area.

## Results

### Description of studies

The six RCTs included in this review provided data on a total of 674 women, with sample sizes ranging from 21 to 196 women. All studies were conducted in the USA. All six studies employed the 17‐item Hamilton Depression Rating Scale (HAMD‐17) as the primary outcome measure for depression response, with some studies also measuring depression using other instruments.

We contacted Sage Therapeutics Inc. to request data for depression response and remission measures at day 30 (as the most distal time point) for two studies (Meltzer‐Brody 2018 Study 1; Meltzer‐Brody 2018 Study 2). We made the request because the primary study publication only presented these data graphically. Sage Therapeutics Inc. supplied the requested data on 28 September 2022. We subsequently requested data for other earlier time points, which were also only presented graphically, but we received no response from the company. We used PlotDigitizer to extract data from Figure 3 of Kanes 2017 and Figures S1 and S2 of Meltzer‐Brody 2018 Study 1 and Meltzer‐Brody 2018 Study 2.

We also corresponded by email with Marinus Pharmaceuticals to confirm the status of two trials registered on ClinicalTrials.gov. We were able to include one trial as the company supplied additional data (NCT03228394), and we excluded the other one (NCT03460756; see details below).

#### Results of the search

We identified 1429 records through database searching, and zero records through additional searching. After we removed duplicates, 650 records remained. We screened the abstracts of 596 records and discarded 561 as they were not relevant. We assessed the eligibility of 35 full‐text articles and excluded 16 (nine studies), with reasons given (see Excluded studies). We determined that 19 reports met inclusion criteria: these reported on six studies which we included in quantitative synthesis/meta‐analysis, and one study awaiting classification (Figure 1). The search results are in Appendix 3 and additional reports of included studies are in Appendix 4.

#### Included studies

##### Participants

All trial inclusion criteria required women to have had a major depressive episode with onset no earlier than the third trimester and no later than four weeks after delivery, a HAMD‐17 total score of 26 or higher (except Meltzer‐Brody 2018 Study 2, which required a HAMD‐17 total score of 20‐25), and to be within six months of childbirth. All six studies excluded women with active suicidal ideation or behaviour, attempted suicide associated with the current episode of PND or a history of bipolar disorder, schizophrenia and/or schizoaffective disorder.

##### Interventions

Three trials tested intravenous brexanolone (Kanes 2017; Meltzer‐Brody 2018 Study 1; Meltzer‐Brody 2018 Study 2), one tested intravenous ganaxolone (NCT03228394), and two tested oral zuranolone (Deligiannidis 2021; Deligiannidis 2023).

Meltzer‐Brody 2018 Study 1 was a three‐arm study of 138 women, where brexanolone was administered at two dosages (60 μg/kg/h and 90 μg/kg/h) and compared with a placebo. Meltzer‐Brody 2018 Study 2 of 108 women administered brexanolone at the higher dose of 90 μg/kg/h only and compared this with a placebo. Both studies administered brexanolone over 60 hours. In Kanes 2017, the dose of brexanolone varied across the 60‐hour infusion (30 μg/kg/h (0 to 4 hours); 60 μg/kg/h (4 to 24 hours); 90 μg/kg/h (24 to 52 hours); 60 μg/kg/h (52 to 56 hours); 30 μg/kg/h (56 to 60 hours)). Details of dosage schedules used in the included studies are reported in Characteristics of included studies.

The study of ganaxolone delivered it intravenously in three participant groups and used a mix of intravenous and oral administration in the fourth group (NCT03228394). Four separate placebo‐matched groups were also studied. In the first group, ganaxolone was administered at a rate of 4 mg/h (16 mL/h) for 48 hours, then 2 mg/h for 12 hours. In group 2, it was at a rate of 8 mg/h for 48 hours, then 4 mg/h (8 mL/h) for 12 hours. For the third group, a bolus of 12 mg (24 mL) was given over two minutes, followed by 12 mg/h (24 mL/h) for 48 hours, then 6 mg/h (12 mL/h) for 12 hours. We did not include data from the fourth group in our meta‐analysis as we stated a priori that we would analyse oral and intravenous drugs separately. For further details, see Characteristics of included studies.

Two studies of 153 and 196 women tested zuranolone, which was administered orally (Deligiannidis 2021; Deligiannidis 2023). Doses ranged from 30 to 50 mg per day, for two weeks.

##### Comparators

All six studies used a placebo control (Deligiannidis 2021; Deligiannidis 2023; Kanes 2017; Meltzer‐Brody 2018 Study 1; Meltzer‐Brody 2018 Study 2; NCT03228394). None of the included studies used another pharmacological intervention, psychological or psychosocial interventions, or treatment as usual as controls.

#### Ongoing studies

There are no ongoing studies.

#### Excluded studies

We excluded nine studies (16 references). In five studies, participants had major depressive disorder rather than PND (Clayton 2020; Gunduz‐Bruce 2019; Hoffmann 2019; NCT03864614; NCT04442490). One study was a phase four study which was withdrawn by the pharmaceutical company (NCT04273191). Riesenberg 2022 was an open‐label study of brexanolone and did not use a comparator. The two remaining excluded studies were not RCTs (NCT03460756; NCT03924492). NCT03460756 initially appeared to be an RCT. When we checked the study results published on ClinicalTrials.gov, we noticed there were no placebo data. We contacted Marinus Pharmaceuticals, who confirmed in correspondence that "after completing 4 open‐label cohorts (those published), the study was stopped and double‐blind, placebo‐controlled trials were never started. However, the title had never been updated to reflect this change". See Characteristics of excluded studies.

### Risk of bias in included studies

As pre‐specified in our protocol, we only included RCTs in this review. RCTs offer the most robust evaluation of the benefits and harms of an intervention. However, methodological shortcomings can give rise to biases that can influence study results. We assessed the risk of such biases in the included studies using the original Cochrane risk of bias tool (Higgins 2011). We present a summary below and in Figure 2. Details for each study can also be found in Figure 3 and the risk of bias tables (Characteristics of included studies).

**2:**
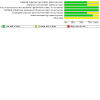
Risk of bias graph: review authors' judgements about each risk of bias item presented as percentages across all included studies.

**3:**
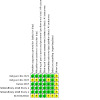
Risk of bias summary: review authors' judgements about each risk of bias item for each included study.

#### Allocation

We judged four studies to be at low risk of selection bias as they used computer‐generated randomisation schedules and described appropriate means of allocation concealment (Deligiannidis 2021; Kanes 2017; Meltzer‐Brody 2018 Study 1; Meltzer‐Brody 2018 Study 2). The remaining two studies did not provide enough detail on randomisation and allocation to permit a judgement (Deligiannidis 2023; NCT03228394).

#### Blinding

##### Performance bias

All six included studies reported adequate blinding of participants and personnel. We judged them to be at low risk of performance bias.

##### Detection bias

All six included studies reported adequate blinding of outcome assessors. We judged them to be at low risk of detection bias.

#### Incomplete outcome data

Four included studies reported dropouts and reasons for discontinuation. We judged them to be at low risk of attrition bias (Deligiannidis 2021; Kanes 2017; Meltzer‐Brody 2018 Study 1; Meltzer‐Brody 2018 Study 2). We deemed Deligiannidis 2023 to be at unclear risk as a higher number of participants in the zuranolone group (21.4% at day 28 compared to 12.4% in the placebo group) were lost to follow‐up or discontinued treatment, so this could have introduced bias. We also judged NCT03228394 to be at unclear risk due to insufficient reporting of dropouts and reasons for discontinuation.

#### Selective reporting

We judged five of the included studies to be at low risk of reporting bias as the protocols were available and all reported the pre‐specified outcomes (Deligiannidis 2021; Deligiannidis 2023; Kanes 2017; Meltzer‐Brody 2018 Study 1; Meltzer‐Brody 2018 Study 2). We judged NCT03228394 to be at unclear risk as there was insufficient information to permit a judgement.

#### Other potential sources of bias

We deemed all six studies to be at unclear risk of other bias due to sponsorship from biopharmaceutical companies. Sage Therapeutics sponsored five (Deligiannidis 2021; Deligiannidis 2023; Kanes 2017; Meltzer‐Brody 2018 Study 1; Meltzer‐Brody 2018 Study 2), and Marinus Pharmaceuticals sponsored NCT03228394. The drug manufacturers thus appear to have had a considerable role in the design and conduct of the studies. Many of the authors also declared financial conflicts of interest as employees or stock owners of the drug manufacturer.

Authors of NCT03228394 published their results on ClinicalTrials.gov only and have not published the results as a peer‐reviewed publication. When extracting data for this trial, we noted that results posted on ClinicalTrials.gov included the outcome severity of depression, measured with HAMD, at day 29 for the intravenous ganaxolone and placebo cohorts. However, we later noticed that the data for this outcome, at this time point, in these six treatment groups, had disappeared, with no record of the results being amended. We contacted ClinicalTrials.gov, who advised us to contact Marinus Pharmaceuticals. Their response was that the change on ClinicalTrials.gov was made to enhance clarity that no participants in these treatment cohorts were analysed at this time point (the statistical analysis plan reports that HAMD‐17 was evaluated at day 29 on cohort 6 only).

### Effects of interventions

See: Table 1; Table 2

We found no studies comparing a neurosteroid GABA_A_ positive allosteric modulator to another active intervention. Thus, there are no data in this version of the review for the following four pre‐specified comparisons of interest.

- Intravenous neurosteroid GABA_A_ positive allosteric modulators versus another pharmacological intervention
- Intravenous neurosteroid GABA_A_ positive allosteric modulators versus any other intervention (e.g. treatment as usual, psychological or psychosocial intervention)
- Oral neurosteroid GABA_A_ positive allosteric modulators versus another pharmacological intervention
- Oral neurosteroid GABA_A_ positive allosteric modulators versus any other intervention (e.g. treatment as usual, psychological or psychosocial intervention)

#### Intravenous neurosteroid GABA_A_ receptor positive allosteric modulators versus placebo

Three studies tested intravenous brexanolone (Kanes 2017; Meltzer‐Brody 2018 Study 1; Meltzer‐Brody 2018 Study 2), and one tested intravenous ganaxolone (NCT03228394). All four studies used a placebo control.

##### Depression response (early phase: < 5 weeks from commencement of treatment)

Four studies measured depression response, defined as at least a 50% reduction in HAMD‐17 total score, in the early phase (Kanes 2017; Meltzer‐Brody 2018 Study 1; Meltzer‐Brody 2018 Study 2; NCT03228394).

At 30 days (the time point closest to the end of the early phase), the meta‐analysis showed that intravenous neurosteroid GABA_A_ receptor positive allosteric modulators may result in little to no difference in depression response (RR 1.24, 95% CI 0.74 to 2.06; P = 0.41; 3 studies, 267 women; low‐certainty evidence; Analysis 1.1). Heterogeneity was high (I^2^ = 78%), due to inconsistency in the effect estimates and confidence intervals in the three studies, which may be related to the differing inclusion criteria of Meltzer‐Brody 2018 Study 2.

Meta‐analysis (Analysis 1.1) showed the following results at earlier time points:

- 2 hours: RR 1.03, 95% CI 0.37 to 2.88; P = 0.95, I^2^ = 0%; 3 studies, 267 women;
- 4 hours: RR 1.13, 95% CI 0.62 to 2.06; P = 0.70, I^2^ = 0%; 3 studies, 267 women;
- 8 hours: RR 1.11, 95% CI 0.70 to 1.74; P = 0.66, I^2^ = 0%; 3 studies, 267 women;
- 12 hours: RR 0.88, 95% CI 0.60 to 1.29; P = 0.51, I^2^ = 0%; 4 studies, 325 women;
- 24 hours: RR 1.17, 95% CI 0.81 to 1.70; P = 0.41, I^2^ = 30%; 4 studies, 325 women;
- 36 hours: RR 1.32, 95% CI 0.99 to 1.77; P = 0.06, I^2^ = 18%; 3 studies, 267 women;
- 48 hours: RR 1.17, 95% CI 0.91 to 1.50; P = 0.23, I^2^ = 25%; 4 studies, 325 women;
- 60 hours: RR 1.27, 95% CI 1.05 to 1.54; P = 0.02, I^2^ = 0%; 4 studies, 325 women;
- 72 hours: RR 1.25, 95% CI 1.03 to 1.51; P = 0.03, I^2^ = 7%; 4 studies, 325 women;
- 7 days: RR 1.34, 95% CI 0.90 to 1.99; P = 0.15, I^2^ = 49%; 3 studies, 267 women.

##### Depression response (acute phase: 5 to 12 weeks from commencement of treatment)

One study measured depression response in the acute phase (NCT03228394). At day 36 post‐infusion, 15 of 26 women responded to ganaxolone treatment compared to 12 out of 25 women given a placebo.

##### Depression response (continuation phase: > 12 weeks from commencement of treatment)

No study measured this outcome at this time point.

##### Depression remission (early phase: < 5 weeks from commencement of treatment)

Four studies measured depression remission (defined as HAMD‐17 total score ≤ 7) at various time points in the early phase (Kanes 2017; Meltzer‐Brody 2018 Study 1; Meltzer‐Brody 2018 Study 2; NCT03228394).

At 30 days (the time point closest to the end of the early phase), the meta‐analysis showed that intravenous neurosteroid GABA_A_ receptor positive allosteric modulators may result in little to no difference in depression remission (RR 1.18, 95% CI 0.59 to 2.38; P = 0.64; 3 studies, 267 women; low‐certainty evidence; Analysis 1.2). Heterogeneity was high (I^2^ = 73%); this was also due to inconsistency in the effect estimates and confidence intervals in the three studies, which may be related to the differing inclusion criteria of Meltzer‐Brody 2018 Study 2.

Meta‐analysis (Analysis 1.2) showed the following results at earlier time points:

- 2 hours: RR 1.00, 95% CI 0.06 to 15.58; 2 studies, 129 women;
- 4 hours: RR 0.80, 95% CI 0.16 to 3.93; P = 0.78, I^2^ = 10%; 2 studies, 129 women;
- 8 hours: RR 1.23, 95% CI 0.18 to 8.48; P = 0.83, I^2^ = 51%; 3 studies, 267 women;
- 12 hours: RR 1.52, 95% CI 0.47 to 4.91; P = 0.48, I^2^ = 23%; 4 studies, 325 women;
- 24 hours: RR 1.60, 95% CI 0.91 to 2.82; P = 0.11, I^2^ = 3%; 4 studies, 325 women;
- 36 hours: RR 1.49, 95% CI 0.92 to 2.42; P = 0.11, I^2^ = 0%; 3 studies, 267 women;
- 48 hours: RR 1.60, 95% CI 1.06 to 2.40; P = 0.02, I^2^ = 0%; 4 studies, 325 women;
- 60 hours: RR 1.68, 95% CI 1.01 to 2.80; P = 0.05, I^2^ = 39%; 4 studies, 325 women;
- 72 hours: RR 1.65, 95% CI 1.19 to 2.28; P = 0.003, I^2^ = 0%; 4 studies, 325 women;
- 7 days: RR 1.45, 95% CI 0.59 to 3.56; P = 0.42, I^2^ = 73%; 3 studies, 267 women.

##### Depression remission (acute phase: 5 to 12 weeks from commencement of treatment)

NCT03228394 did not measure depression remission in the acute phase.

##### Depression remission (continuation phase: > 12 weeks from commencement of treatment)

No study measured this outcome at this time point.

##### Adverse events (mother)

The included studies reported adverse events such as somnolence, nausea and vomiting, diarrhoea, dizziness and headache (Kanes 2017; Meltzer‐Brody 2018 Study 1; Meltzer‐Brody 2018 Study 2; NCT03228394). Meta‐analysis suggested that intravenous neurosteroid GABA_A_ positive allosteric modulators are probably associated with little or no difference in the number of adverse events compared to placebo (RR 1.02, 95% CI 0.71 to 1.48; P = 0.90, I^2^ = 46%; 4 studies, 325 women; moderate‐certainty evidence; Analysis 1.3).

No deaths occurred in any of the included studies (Kanes 2017; Meltzer‐Brody 2018 Study 1; Meltzer‐Brody 2018 Study 2; NCT03228394). Three studies reported on severe adverse events (Kanes 2017; Meltzer‐Brody 2018 Study 1; Meltzer‐Brody 2018 Study 2): these were somnolence, fatigue, presyncope and loss of consciousness (Meltzer‐Brody 2018 Study 1; Meltzer‐Brody 2018 Study 2). Meta‐analysis comparing with placebo showed a risk ratio of 1.17 (95% CI 0.23 to 5.89; P = 0.85, I^2^ = 0%; 267 women; Analysis 1.3). Serious adverse events were also measured in four trials (Kanes 2017; Meltzer‐Brody 2018 Study 1; Meltzer‐Brody 2018 Study 2; NCT03228394), and consisted of suicidal ideation and attempts (Meltzer‐Brody 2018 Study 1), and altered consciousness and syncope (Meltzer‐Brody 2018 Study 2). Meta‐analysis comparing with placebo showed a risk ratio of 2.13 (95% CI 0.23 to 20.21; P = 0.51, I^2^ = 0%; 325 women; Analysis 1.3).

##### Adverse events (nursing baby)

None of the studies reported any adverse events in the nursing infant.

##### Severity of depression (early phase: < 5 weeks from commencement of treatment)

At 30 days, the time point closest to the end of the early phase, the meta‐analysis showed that intravenous neurosteroid GABA_A_ receptor positive allosteric modulators may result in little to no difference in the severity of PND (MD ‐4.22, 95% CI ‐8.46 to 0.02; P = 0.05; 3 studies, 267 women; low‐certainty evidence; Analysis 1.4), measured by HAMD‐17. However, heterogeneity was high (I^2^ = 78%), which may be related to the differing inclusion criteria of Meltzer‐Brody 2018 Study 2.

Results for the severity of PND at earlier timepoints are presented in Analysis 1.4 and are as follows:

- 2 hours: MD ‐0.29, 95% CI ‐1.22 to 0.64; P = 0.54, I^2^= 0%; 3 studies, 267 women;
- 4 hours: MD ‐1.12, 95% CI ‐2.30 to 0.06; P = 0.06, I^2^= 0%; 3 studies, 267 women;
- 8 hours: MD ‐1.27, 95% CI ‐2.56 to 0.03; P = 0.06, I^2^ = 0%; 3 studies, 267 women;
- 12 hours: MD ‐0.45, 95% CI ‐1.88 to 0.97; P = 0.53, I^2^ = 22%; 4 studies, 325 women;
- 24 hours: MD ‐2.96, 95% CI ‐5.26 to ‐0.66; P = 0.01, I^2^ = 56%; 4 studies, 325 women;
- 36 hours: MD ‐3.7, 95% CI ‐6.62 to ‐0.79; P = 0.01, I^2^ = 66%; 3 studies, 267 women;
- 48 hours: MD ‐3.77, 95% CI ‐5.91 to ‐1.64; P = 0.0005, I^2^ = 40%; 4 studies, 325 women;
- 60 hours: MD ‐3.75, 95% CI ‐6.13 to ‐1.37; P = 0.002, I^2^ = 57%; 4 studies, 325 women;
- 72 hours: MD ‐3.64, 95% CI ‐6.00 to ‐1.28; P = 0.003, I^2^ = 50%; 4 studies, 325 women;
- 7 days: MD ‐4.11, 95% CI ‐7.08 to ‐1.14; P = 0.007, I^2^ = 58%; 3 studies, 267 women.

In addition, at 11 days, for NCT03228394, the mean difference was ‐1.00 (95% CI ‐6.17 to 4.17; 1 study, 58 women).

Kanes 2017 also used the Montgomery‐Åsberg Depression Rating Scale (MADRS) in the early phase:

- 24 hours: MD ‐17.5 (SE 5.4), P = 0.0042;
- 48 hours: MD ‐18.4 (5.3), P = 0.0026;
- 60 hours: MD ‐15.9 (5.5), P = 0.0104;
- 72 hours: MD ‐16.2 (5.5), P = 0.0090;
- 7 days: MD ‐16.0 (5.4), P = 0.0091;
- 30 days: MD ‐15.1 (5.2), P = 0.0100.

Meltzer‐Brody 2018 Study 1 and Meltzer‐Brody 2018 Study 2 also used the MADRS as follows:

- Meltzer‐Brody 2018 Study 1 at 60 hours: 60 μg/kg/hour least squared (LS) mean difference (MD) ‐6.9 (SE 2.4), P = 0.0054; 90 μg/kg/hour LS MD ‐4.2 (2.4), P = 0.0763;
- Meltzer‐Brody 2018 Study 1 at 30 days: 60 μg/kg/hour LS MD ‐5.6 (2.8), P = 0.0447; 90 μg/kg/hour LS MD ‐3.6 (2.7), P = 0.1908;
- Meltzer‐Brody 2018 Study 2 at 60 hours: LS MD ‐4.9 (SE 1.6), P = 0.0033;
- Meltzer‐Brody 2018 Study 2 at 30 days: LS MD 0.0 (SE 1.8), P = 0.9845.

Meltzer‐Brody 2018 Study 1 and Meltzer‐Brody 2018 Study 2 also used the Edinburgh Postnatal Depression Scale (EPDS) as follows:

- Meltzer‐Brody 2018 Study 1 at 60 hours: 60 μg/kg/hourLS MD ‐1.6 (SE 1.4), P = 0.2531; 90 μg/kg/hour LS MD ‐1.1 (1.4), P = 0.4202;
- Meltzer‐Brody 2018 Study 1 at 30 days: 60 μg/kg/hour LS MD ‐3.7 (1.7), P = 0.0290; 90 μg/kg/hour LS MD ‐1.8 (1.6), P = 0.1908;
- Meltzer‐Brody 2018 Study 2 at 60 hours: LS MD ‐1.8 (SE 1.2), P = 0.1320;
- Meltzer‐Brody 2018 Study 2 at 30 days: LS MD 0.4 (SE 1.2), P = 0.7158.

Meltzer‐Brody 2018 Study 1 and Meltzer‐Brody 2018 Study 2 also used the Patient Health Questionnaire (PHQ‐9) as follows:

- Meltzer‐Brody 2018 Study 1 at 60 hours: 60 μg/kg/hour LS MD ‐0.9 (SE 1.6), P = 0.5688; 90 μg/kg/hour LS MD ‐0.9 (1.5), P = 0.5464;
- Meltzer‐Brody 2018 Study 1 at 30 days: 60 μg/kg/hour LS MD ‐2.5 (1.6), P = 0.1305; 90 μg/kg/hour LS MD ‐2.4 (1.6), P = 0.1331;
- Meltzer‐Brody 2018 Study 2 at 60 hours: LS MD ‐1.2 (SE 1.3), P = 0.3764;
- Meltzer‐Brody 2018 Study 2 at 30 days: LS MD ‐0.5 (SE 1.1), P = 0.6912.

NCT03228394 reported EPDS at days 3, 11 and 34.

- At day 3, the mean change in EPDS was ‐10.37 (SD 6.49) in 30 women randomised to intravenous ganaxolone compared to ‐11.01 (SD 7.7) in 28 women randomised to a placebo.
- At day 11, the mean change in EPDS was ‐9.82 (SD 7.29) in 30 women randomised to intravenous ganaxolone compared to ‐9.87 (SD 7.43) in 28 women randomised to a placebo.
- At day 34, the mean change in EPDS was ‐11.07 (SD 6.86) in 30 women randomised to intravenous ganaxolone compared to ‐10.22 (SD 6.51) in 28 women randomised to a placebo.

##### Severity of depression (acute phase: 5 to 12 weeks from commencement of treatment)

NCT03228394 measured severity of depression at 36 days. In 30 women randomised to ganaxolone, HAMD‐17 severity was reduced by ‐13.57 (SD 10.74) while in 28 women randomised to a placebo, mean severity was reduced by ‐12.21 (SD 8.58).

##### Severity of depression (continuation phase: > 12 weeks from commencement of treatment)

No study measured this outcome at this time point.

##### Treatment acceptability

Three studies reported on study dropouts (Kanes 2017; Meltzer‐Brody 2018 Study 1; Meltzer‐Brody 2018 Study 2). Meta‐analysis suggested that intravenous neurosteroid GABA_A_ positive allosteric modulators are probably associated with an increased number of dropouts compared to placebo (RR 2.77, 95% CI 1.22 to 6.26; P = 0.01, I^2^ = 0%; 3 studies, 267 women; moderate‐certainty evidence).

##### Quality of life

No study measured this outcome.

##### Parenting‐ and child‐related outcomes

No study measured this outcome.

#### Oral neurosteroid GABA_A_ positive allosteric modulators versus placebo

Two studies compared oral zuranolone versus placebo control (Deligiannidis 2021; Deligiannidis 2023).

##### Depression response (early phase: < 5 weeks from commencement of treatment)

Both Deligiannidis 2021 and Deligiannidis 2023 measured depression response as at least a 50% reduction in HAMD‐17 total score. This outcome was measured at days 3, 8, 15 and 21 in both studies and also at day 28 in Deligiannidis 2023. Meta‐analysis showed that zuranolone was associated with a depression response until day 21 (Analysis 2.1):

- Day 3: RR 1.70, 95% CI 1.17 to 2.47; P = 0.006; I^2^ = 0%, 2 studies, 349 women;
- Day 8: RR 1.64, 95% CI 1.24 to 2.18; P = 0.006; I^2^ = 23%, 2 studies, 349 women;
- Day 15: RR 1.50, 95% CI 1.21 to 1.86; P = 0.0002; I^2^ = 0%, 2 studies, 349 women;
- Day 21: RR 1.33, 95% CI 1.09 to 1.63; P = 0.006; I^2^ = 0%, 2 studies, 349 women;
- Day 28: RR 1.37, 95% CI 0.98 to 1.91; P = 0.06; 1 study, 196 women.

##### Depression response (acute phase: 5 to 12 weeks from commencement of treatment)

Both studies also measured depression response at day 45 (Deligiannidis 2021; Deligiannidis 2023). Moderate‐certainty evidence showed that zuranolone is probably associated with a HAMD‐17 response in the acute phase (RR 1.26, 95% CI 1.03 to 1.55; P = 0.03, I^2^ = 13%; 2 studies, 349 women; Analysis 2.2).

##### Depression response (continuation phase: > 12 weeks from commencement of treatment)

Neither study measured this outcome at this time point.

##### Depression remission (early phase: < 5 weeks from commencement of treatment)

Both Deligiannidis 2021 and Deligiannidis 2023 measured depression remission as achieving a HAMD‐17 total score of 7 or less. This outcome was measured at days 3, 8, 15 and 21 in both studies and at day 28 in Deligiannidis 2023. Analysis 2.3 showed that zuranolone was associated with remission of depression until day 21:

- Day 3: RR 2.37, 95% CI 1.11 to 5.05; P = 0.03, I^2^ = 0%; 2 studies, 349 women;
- Day 8: RR 1.63, 95% CI 1.04 to 2.55; P = 0.03, I^2^ = 0%; 2 studies, 349 women;
- Day 15: RR 1.81, 95% CI 1.24 to 2.63; P = 0.002, I^2^ = 0%; 2 studies, 349 women;
- Day 21: RR 1.46, 95% CI 1.03 to 2.08; P = 0.03, I^2^ = 0%; 2 studies, 349 women;
- Day 28: RR 1.30, 95% CI 0.78 to 2.17; P = 0.31; 1 study, 196 women.

##### Depression remission (acute phase: 5 to 12 weeks from commencement of treatment)

Both studies measured depression remission at day 45. Moderate‐certainty evidence showed that zuranolone is probably associated with depression remission in the acute phase (RR 1.65, 95% CI 1.22 to 2.22; P = 0.001, I^2^ = 0%; 2 studies, 349 women; Analysis 2.4).

##### Depression remission (continuation phase: > 12 weeks from commencement of treatment)

No study measured this outcome at this time point.

##### Adverse events (mother)

Moderate‐certainty evidence showed that zuranolone is probably associated with more maternal adverse events (RR 1.24, 95% CI 1.03 to 1.48; P = 0.02, I^2^ = 0%; 2 studies, 349 women; moderate‐certainty evidence; Analysis 2.5).

Both studies measured severe adverse events, including sedation, dizziness and headache. Meta‐analysis showed a risk ratio of 1.42 (95% CI 0.39 to 5.14; P = 0.59, I^2^ = 0%; 2 studies, 349 women; Analysis 2.5).

Both studies also measured serious adverse events. Meta‐analysis showed a risk ratio of 2.06 (95% CI 0.27 to 15.77; P = 0.49, I^2^ = 0%; 2 studies, 349 women; Analysis 2.5). In Deligiannidis 2021, one serious adverse event was reported in each arm. One participant in the zuranolone group experienced a confusional state and sedation on day 3, which resolved within seven hours. The dose was reduced to 20 mg the following day and the patient continued treatment without further incident. One participant on placebo had pancreatitis on day 32 of follow‐up, which resolved on day 36 with cholecystectomy. In Deligiannidis 2023, two serious adverse events were reported, both of them in participants in the zuranolone group. One was during the treatment course and one during the post‐treatment period and were considered unrelated to the study drug. No loss of consciousness and no clinically significant changes in vital signs, electrocardiogram or clinical laboratory parameters were reported.

No deaths occurred in either of the included studies (Deligiannidis 2021; Deligiannidis 2023).

##### Adverse events (nursing baby)

Neither of the studies reported any adverse events in the nursing infant.

##### Severity of depression (early phase: < 5 weeks from commencement of treatment)

Both studies reported least squares mean difference in HAMD‐17 total score at days 3, 8, 15 and 21. Deligiannidis 2023 also measured this outcome at day 28. Analysis 2.6 showed the following:

- Day 3: LS MD ‐3.10, 95% CI ‐4.62 to ‐1.59; P < 0.001, I^2^ = 0%; 2 studies, 349 women;
- Day 8: LS MD ‐3.58, 95% CI ‐5.20 to ‐1.96; P < 0.001, I^2^ = 0%; 2 studies, 349 women;
- Day 15: LS MD ‐4.08, 95% CI ‐5.83 to ‐2.34; P < 0.001, I^2^ = 0%; 2 studies, 349 women;
- Day 21: LS MD ‐2.75, 95% CI ‐4.58 to ‐0.93; P = 0.003, I^2^ = 0%; 2 studies, 349 women;
- Day 28: LS MD ‐2.90, 95% CI ‐5.35 to ‐0.45; P = 0.02; 1 study, 196 women.

In addition to the study's primary outcome of HAMD‐17, Deligiannidis 2023 reported the least squares mean difference from baseline in the MADRS score at the following time points:

- Day 3: LS MD ‐4.6, 95% CI ‐7.7 to ‐1.5; P = 0.004;
- Day 15: LS MD ‐5.1, 95% CI ‐8.4 to ‐1.7; P = 0.003;
- Day 28: LS MD ‐3.4, 95% CI ‐6.8 to 0; P = 0.051.

MADRS was also reported in Deligiannidis 2021 at day 15 (MD −4.6, 95% CI −8.3 to −0.8; P = 0.02).

Change from baseline EPDS (as least squares mean difference) was also reported by Deligiannidis 2023 as follows:

- Day 3: LS MD ‐1.5, 95% CI ‐2.9 to ‐0.1; P = 0.03;
- Day 8: LS MD ‐2.2, 95% CI ‐3.8 to ‐0.5; P = 0.01;
- Day 15: LS MD ‐2.0, 95% CI ‐3.8 to ‐0.1; P = 0.04.

##### Severity of depression (acute phase: 5 to 12 weeks from commencement of treatment)

Both studies measured the severity of depression at day 45. Meta‐analysis showed that zuranolone is probably associated with an improvement in the HAMD‐17 score from baseline (MD ‐3.79, 95% CI ‐5.60 to ‐1.97; P < 0.001, I^2^ = 0%; 349 women; moderate‐certainty evidence; Analysis 2.7).

The least squares mean difference from baseline in MADRS was reported at day 45 by Deligiannidis 2021 (LS MD ‐5.8, 95% CI ‐9.4 to ‐2.2; P = 0.002).

Deligiannidis 2023 also reported the least squares mean difference from baseline in the MADRS score at day 45 (LS MD ‐4.7, 95% CI ‐8.3 to ‐1.1; P = 0.010) and the EPDS score at day 45 (LS MD ‐2.4, 95% CI ‐4.5 to ‐0.3; P = 0.03).

##### Severity of depression (continuation phase: > 12 weeks from commencement of treatment)

No study measured this outcome at this time point.

##### Treatment acceptability

Low‐certainty evidence showed little to no difference in treatment acceptability, as measured by the number of study dropouts (RR 0.95, 95% CI 0.50 to 1.81; P = 0.88; I² = 5%; 2 studies, 349 women; Analysis 2.8).

##### Quality of life

No study measured this outcome.

##### Parenting‐ and child‐related outcomes (early phase: < 5 weeks from commencement of treatment)

Deligiannidis 2021 measured the Barkin Index of Maternal Functioning (BIMF): a validated measure of patient‐reported maternal function within the first year of childbirth, where a higher score indicates better functioning. Authors reported the least squares mean difference from baseline at the following timepoints (Analysis 2.9):

- Day 3: LS MD 2.2, 95% CI ‐1.19 to 5.59; P = 0.20;
- Day 8: LS MD 1.1, 95% CI ‐3.19 to 5.39; P = 0.62;
- Day 15: LS MD 4.7, 95% CI ‐0.75 to 10.15; P = 0.09;
- Day 21: LS MD 3.6, 95% CI ‐1.89 to 9.09; P = 0.20.

##### Parenting‐ and child‐related outcomes (early phase: < 5 weeks from commencement of treatment)

Deligiannidis 2021 reported that women who received zuranolone had a higher BIMF score at day 45 than those given a placebo (MD 7.20, 95% CI 1.42 to 12.98; low‐certainty evidence; Analysis 2.10).

#### Subgroup analysis

For comparison 1, we grouped together three studies on brexanolone and one study on ganaxolone. We were able to conduct subgroup analyses for five outcomes (depression response, depression remission, adverse events, severity of depression and treatment acceptability) looking at brexanolone in isolation. The results of the subgroup analysis resulted in minimal changes when NCT03228394 was excluded. For depression response in the early phase (at 30 days), the risk ratio was 1.24 (95% CI 0.74 to 2.06; P = 0.41, I^2^ = 78%; 267 women; Analysis 3.1). For depression remission at day 30, the risk ratio was 1.18 (95% CI 0.59 to 2.38; P = 0.64, I^2^ = 73%; 267 women; Analysis 3.2). Regarding adverse events, the risk ratio for any adverse events with brexanolone compared to placebo was 0.93 (95% CI 0.71 to 1.21; P = 0.57, I^2^ = 0%; 267 women; Analysis 3.3). The mean difference in HAMD‐17 score as a measure of severity in the early phase (at 30 days) was ‐4.22 (95% CI ‐8.46 to 0.02; P = 0.05, I^2^ = 78%; 267 women; Analysis 3.4). Regarding acceptability, the risk ratio for dropout with brexanolone was 2.77 (95% CI 1.22 to 6.26; P = 0.01, I^2^ = 0%; 267 women; Analysis 3.5).

#### Sensitivity analysis

Due to lack of data, we were not able to perform our planned sensitivity analyses.

## Discussion

### Summary of main results

We identified six eligible studies (674 women) that assessed the benefits and harms of neurosteroid GABA_A_ receptor positive allosteric modulators for the treatment of PND. Three of these compared brexanolone with placebo, one ganaxolone with placebo, and two zuranolone with placebo. All studies measured symptoms of depression using the HAMD‐17 scale. All studies were conducted in the USA.

Three trials (267 women) tested IV brexanolone (Kanes 2017; Meltzer‐Brody 2018 Study 1; Meltzer‐Brody 2018 Study 2), one tested IV ganaxolone (58 women; NCT03228394), and two (349 women) tested oral zuranolone (Deligiannidis 2021; Deligiannidis 2023).

We found that intravenous neurosteroid GABA_A_ receptor positive allosteric modulators (including brexanolone) may be associated with little or no improvements in depression remission, response or severity compared to placebo, when measured at 30 days from commencement of treatment. There is probably little or no difference in maternal adverse events, but treatment acceptability is probably lower than with placebo.

We found that oral neurosteroid GABA_A_ positive allosteric modulators (i.e. zuranolone) are probably associated with greater depression response and remission compared to placebo, when measured at 45 days from commencement of treatment. They probably increase the rate of maternal adverse events (when all adverse events are considered), but make little or no difference to severe and serious adverse events. Moreover, there may be little or no difference in treatment acceptability compared to placebo. They are probably effective in reducing the severity of depression and may result in an improvement in maternal functioning.

### Overall completeness and applicability of evidence

There was no evidence for the benefits and harms of neurosteroid GABA_A_ receptor positive allosteric modulators compared with other pharmacological interventions, treatment as usual, or psychological or psychosocial interventions, so the relative effectiveness compared with other treatments is not yet known. There were also no data on outcomes beyond 45 days from the commencement of treatment, so longer‐term treatment outcomes are also unknown. Safety data related only to women, with no data pertaining to safety for the breastfeeding infant, as participants were asked not to breastfeed while receiving the treatment. No other child‐related outcomes were reported, with only one study measuring parenting functioning. There were also no maternal quality of life‐related outcome measures used.

None of the studies reported direct evidence regarding treatment acceptability; that is, evidence obtained by asking participating women directly about their treatment experience. This is particularly important given that brexanolone is administered as an intravenous infusion under medical supervision, unlike traditional antidepressant medication.

Many of the studies excluded women with suicide attempts and common comorbidities, such as substance use and physical ill health. This may limit the applicability of the evidence to these women.

All six included studies were conducted in the USA, so the global applicability of our findings may be limited. There remains an evidence gap in studies of PND treatment for low‐ and middle‐income countries.

### Certainty of the evidence

We judged the risks of selection, performance, detection, attrition and reporting biases to be low for four studies (Deligiannidis 2021; Kanes 2017; Meltzer‐Brody 2018 Study 1; Meltzer‐Brody 2018 Study 2). Two studies provided insufficient information to judge the risk of selection bias (Deligiannidis 2023; NCT03228394). The studies of brexanolone and zuranolone involved many of the same authors and were conducted by the same pharmaceutical company. Many of the authors declared financial conflicts of interest as employees or stock owners of the drug manufacturer.

For intravenous neurosteroid GABA_A_ receptor positive allosteric modulators, there was low‐certainty evidence for all outcomes assessed, except adverse events and treatment acceptability, for which the evidence was of moderate certainty. The certainty of the evidence was low due to inconsistency and imprecision, with many of the confidence intervals including no effect. The certainty of the evidence for zuranolone was moderate for all outcomes assessed, except maternal functioning and treatment acceptability, for which the evidence was of low certainty.

### Potential biases in the review process

We employed a thorough search strategy to provide a comprehensive synthesis of the evidence to date. However, we could not assess publication bias through a funnel plot analysis due to an insufficient number of studies; thus, publication bias may have influenced the review's findings. Moreover, I^2^ for some of the meta‐analyses, particularly those for the intravenous comparison, suggested considerable heterogeneity. We planned in our protocol to conduct a number of sensitivity and subgroup analyses to explore sources of variation within our results (Wilson 2021). However, due to the small number of included studies, this was only possible for one of the subgroup analyses (of brexanolone).

### Agreements and disagreements with other studies or reviews

A meta‐analysis of three of the studies included in this review – Kanes 2017; Meltzer‐Brody 2018 Study 1; Meltzer‐Brody 2018 Study 2 – reported pooled response and remission rates at day 30 (what we have called in our review the ‘early’ phase) that differed from those in our review (Gerbasi 2021). Having contacted Sage Therapeutics about this difference, the data used in the Gerbasi 2021 meta‐analysis appear to relate to a range of follow‐up time points being chosen for the pooled effect estimates. It was unclear from our correspondence with Sage which time points Gerbasi and colleagues used in their meta‐analysis.

A network meta‐analysis (also sponsored by Sage Therapeutics) of 26 studies indirectly compared the effectiveness of brexanolone to SSRIs, and reported a greater change‐from‐baseline depression score at day 30 for brexanolone than for SSRIs (Cooper 2019).

## Authors' conclusions

**Implications for practice**

British guidelines recommend that postnatal depression (PND) be managed according to the severity of the disease, with traditional antidepressants (such as selective serotonin reuptake inhibitors) being recommended for women with more severe depression, with or without combined treatment with psychological therapy (McAllister‐Williams 2017; NICE 2020). However, there is increasing interest in developing and evaluating novel treatments for mood disorders, including PND, for those who do not respond to traditional antidepressants. There is also interest in addressing the delayed therapeutic response seen with traditional antidepressants.

We found that there are probably benefits of a two‐week course of zuranolone in terms of greater depression response and remission and reduced severity of depression compared to placebo, when measured at 45 days after treatment commencement. We found that there may be little or no difference in response, remission and severity of depression with a 60‐hour course of intravenous neurosteroid GABA_A_ receptor positive allosteric modulators, such as brexanolone, when measured at 30 days after treatment. There is probably an increased rate of maternal adverse events with zuranolone (when all adverse events are considered), but little or no difference in severe and serious adverse events. It is worth noting the practical and financial challenges to using these medications, particularly intravenous formulations, which require hospital admission and insurance approval.

There remain a number of unanswered questions that have implications for the use of neurosteroid GABA_A_ receptor positive allosteric modulators in clinical practice. A key limitation is the lack of evidence comparing these medications to any active intervention, whether antidepressants, psychological therapy or psychosocial interventions. There is also a need to establish the optimal patient population for these medications. While the indication for their use is more severe PND, existing studies exclude women with more severe disease with suicidality and comorbidities.

As in all areas of clinical practice, patient preference remains an important consideration and being guided by patient preference may improve outcomes. Whilst a qualitative interview study of 10 women receiving brexanolone reported that it was generally well accepted (Salwan 2023), there were no data in the included studies on quality of life measures.

**Implications for research**

The current evidence base is limited to a small number of randomised controlled trials on the benefits and harms of neurosteroid GABA_A_ receptor positive allosteric modulators compared to placebo up to 45 days from the commencement of treatment. The focus of future research should be on expanding the evidence base to examine longer‐term outcomes, and comparing these medications not only to placebo but to well‐established treatments, including antidepressants and psychological therapy. Future studies could also usefully include women with more severe PND; for example, those with suicidality. This population may benefit most from a more rapid response to treatment than that achieved with traditional antidepressants. Safety during breastfeeding should be examined. Untreated persistent PND can be associated with adverse child and parenting outcomes, so future studies should include both child and parenting outcome measures. Questions also remain about patient acceptability, particularly of oral neurosteroid GABA_A_ positive allosteric modulators, such as zuranolone.

## What's new

**Table:** 

**Date**	**Event**	**Description**
20 August 2025	Amended	The Results section has been updated in relation to the studies of intravenous brexanolone versus placebo to clarify that Meltzer‐Brody 2018 Study 2 had different inclusion criteria based on the HAMD‐17 score to the other studies in this comparison, and that this may have been a potential reason for heterogeneity in analyses 1.1, 1.2, and 1.4.

## History

Protocol first published: Issue 5, 2021 Review first published: Issue 6, 2025

## Appendices

### Appendix 1. A note regarding the use of the term 'woman'

We have used the term 'woman' to refer to all those who could find themselves pregnant and in the postnatal period. We recognise that this could include individuals with diverse gender identities.

### Appendix 2. Search strategies

**Cochrane Central Register of Controlled Trials (CENTRAL)** (current issue)

#1 (Brexanolone or Zulresso or SAGE‐547 or Ganaxolone or CCD‐1042 or Zuranolone or SAGE‐217)

#2 Allopregnanolone

#3 ((neurosteroid* or (neuro* NEXT steroid*) or (neuroactive NEXT steroid*) or (positive NEXT allosteric NEXT modulat*) or PAM or PAMs)) and ((GABA* or "gamma aminobutyric acid") and receptor*)

#4 (#2 or #3)

#5 ((postpartum* or (post NEXT partum*) or postnatal* or (post NEXT natal*) or perinatal* or (peri NEXT natal*) or puerp* or intrapartum* or (intra NEXT partum*) or antepartum* or (ante NEXT partum*)) and (depress* or dysthymi* or (adjustment NEXT disorder*) or (mood NEXT disorder*) or (affective NEXT disorder*) or (affective NEXT symptom*)))

#6 (#4 and #5)

#7 (#1 or #6) Date added to CENTRAL trials database 10/03/22‐24/01/2024

***************************

**Ovid MEDLINE(R) ALL** <1946 onwards> Search Strategy: ‐‐‐‐‐‐‐‐‐‐‐‐‐‐‐‐‐‐‐‐‐‐‐‐‐‐‐‐‐‐‐‐‐‐‐‐‐‐‐‐‐‐‐‐‐‐‐‐‐‐‐‐‐‐‐‐‐‐‐‐‐‐‐‐‐‐‐‐‐‐‐‐‐‐‐‐‐‐‐‐ 1 (Brexanolone or Zulresso or SAGE‐547 or Ganaxolone or CCD‐1042 or Zuranolone or SAGE‐217).mp. 2 Allopregnanolone.mp. 3 ((neurosteroid* or neuro* steroid* or neuroactive steroid* or positive allosteric modulat* or PAM?) and ((GABA* or gamma aminobutyric acid) and receptor*)).mp. 4 or/1‐3 5 ((postpartum* or post partum* or postnatal* or post natal* or perinatal* or peri natal* or puerp* or intrapartum* or intra partum* or antepartum* or ante partum*) and (depress* or dysthymi* or adjustment disorder* or mood disorder* or affective disorder* or affective symptom*)).mp. 6 (4 and 5) 7 exp animals/ not humans.sh. 8 (6 not 7)

***************************

**Ovid Embase** <1980 onwards> Search Strategy: ‐‐‐‐‐‐‐‐‐‐‐‐‐‐‐‐‐‐‐‐‐‐‐‐‐‐‐‐‐‐‐‐‐‐‐‐‐‐‐‐‐‐‐‐‐‐‐‐‐‐‐‐‐‐‐‐‐‐‐‐‐‐‐‐‐‐‐‐‐‐‐‐‐‐‐‐‐‐‐‐ 1 Brexanolone/ 2 Ganaxolone/ 3 Zuranolone/ 4 (Brexanolone or Zulresso or SAGE‐547 or Ganaxolone or CCD‐1042 or Zuranolone or SAGE‐217).mp. 5 Allopregnanolone.mp.  6 ((neurosteroid* or neuro* steroid* or neuroactive steroid* or positive allosteric modulat* or PAM?) and ((GABA* or gamma aminobutyric acid) and receptor*)).mp. 7 exp 4 aminobutyric acid A receptor stimulating agent/ 8 *GABAergic receptor affecting agent/ 9 or/1‐8 10 postnatal depression/ 11 ((postpartum* or post partum* or postnatal* or post natal* or perinatal* or peri natal* or puerp* or intrapartum* or intra partum* or antepartum* or ante partum*) adj3 (depress* or dysthymi* or adjustment disorder* or mood disorder* or affective disorder* or affective symptom*)).ti,ab,kw. 12 (10 or 11) 13 (9 and 12) 14 ((animal or nonhuman) not human).de. 15 (13 not 14)

***************************

**Ovid APA PsycInfo** <all available years> Search Strategy: ‐‐‐‐‐‐‐‐‐‐‐‐‐‐‐‐‐‐‐‐‐‐‐‐‐‐‐‐‐‐‐‐‐‐‐‐‐‐‐‐‐‐‐‐‐‐‐‐‐‐‐‐‐‐‐‐‐‐‐‐‐‐‐‐‐‐‐‐‐‐‐‐‐‐‐‐‐‐‐‐

1 (Brexanolone or Zulresso or SAGE‐547 or Ganaxolone or CCD‐1042 or Zuranolone or SAGE‐217).mp. 2 Allopregnanolone.mp. 3 ((neurosteroid* or neuro* steroid* or neuroactive steroid* or positive allosteric modulat* or PAM?) and ((GABA* or gamma aminobutyric acid) and receptor*)).mp. 4 (2 or 3) 5 ((postpartum* or "post partum*" or postnatal* or "post natal*" or perinatal* or "peri natal*" or puerp* or intrapartum* or "intra partum*" or antepartum* or "ante partum*") and (depress* or dysthymi* or "adjustment disorder*" or "mood disorder*" or "affective disorder*" or "affective symptom*")).mp.  6 (4 and 5) 7 (1 or 6)

***************************

**ClinicalTrials.gov** <all available years> Search Strategy: ‐‐‐‐‐‐‐‐‐‐‐‐‐‐‐‐‐‐‐‐‐‐‐‐‐‐‐‐‐‐‐‐‐‐‐‐‐‐‐‐‐‐‐‐‐‐‐‐‐‐‐‐‐‐‐‐‐‐‐‐‐‐‐‐‐‐‐‐‐‐‐‐‐‐‐‐‐‐‐‐

Brexanolone OR Zulresso OR SAGE‐547 First posted from 03/10/2022 to 01/24/2024 (manually screened for PND)

Ganaxolone OR CCD‐1042 First posted from 03/10/2022 to 01/24/2024 (screened for PND)

Zuranolone OR SAGE‐217 First posted from 03/10/2022 to 01/24/2024 (screened for PND)

***************************

**WHO ITCRP** <all available years> Search Strategy: ‐‐‐‐‐‐‐‐‐‐‐‐‐‐‐‐‐‐‐‐‐‐‐‐‐‐‐‐‐‐‐‐‐‐‐‐‐‐‐‐‐‐‐‐‐‐‐‐‐‐‐‐‐‐‐‐‐‐‐‐‐‐‐‐‐‐‐‐‐‐‐‐‐‐‐‐‐‐‐‐

Brexanolone OR Zulresso OR SAGE‐547 Date of registration is between 10/03/2022 and 24/01/2024 (manually screened for PND)

Ganaxolone OR CCD‐1042 Date of registration is between 10/03/2022 and 24/01/2024 (screened for PND)

Zuranolone OR SAGE‐217 Date of registration is between 10/03/2022 and 24/01/2024 (screened for PND)

### Appendix 3. Search results

**Search 4**

Date of search: 24 January 2024

CLib:CENTRAL <Issue 1, January 2024 >, n=55

Ovid MEDLINE(R) ALL <1946 to January 23, 2024>, n=48

Ovid APA PsycInfo <January 2024>, n=33

Ovid Embase <1996 to 2024, week 3>, n=162

ClinicalTrials.gov (0)

WHO‐ICTRP (1)

Total=299

Total after software de‐duplication = 219

**Search‐3**

Date of search: 10 March 2022

Date of search: 10 March 2022

CLib:CENTRAL <Issue 3, March 2022>, n=15

Ovid MEDLINE(R) ALL <2021 to March 10, 2022>, n=36

Ovid APA PsycInfo <2021 to March 10, 2022>, n=26

Ovid Embase <2021 to March 10, 2022>, n=77

ClinicalTrials.gov (2)

WHO‐ICTRP (11)

Total=167

Duplicates removed within this set, n=58 [109]

Duplicates removed from search‐1 & search‐2, n=49

Additional records to screen, n=60

**Search‐2**

Date of search: 14 May 2021

CLib:CENTRAL <Issue 5, May 2021>, n=122

Ovid MEDLINE(R) ALL <1946 to May 14, 2021>, n=108

Ovid APA PsycInfo <1806 to May Week 2 2021>, n=109

Ovid Embase <1980 to 2021 Week 19>, n=224

ClinicalTrials.gov (4)

Total=567

Duplicates removed (within this set and from Search‐1), n=411

Additional records to screen, n=156

**Search‐1**

Date of search: 9 January 2021

• Cochrane Library, Issue 1 of 12, 2021 n=55

• Ovid MEDLINE 1946 to 7 Jan 2021, n=101

• Ovid PsycINFO all years to January Week 1 2021, n=55

• Ovid Embase 1980 to 2021 Week 01, n=172

• ClinicalTrials.gov (all years), n=12

• Drugs@FDA, n=1

Total=396

Duplicates removed, n= 165

Records to screen, n=231

***************************************

**Ovid Embase** <1974 to 2022 March 10>

1 Brexanolone/ 662

2 Ganaxolone/ 410

3 Zuranolone/ 71

4 (Brexanolone or Zulresso or SAGE‐547 or Ganaxolone or CCD‐1042 or Zuranolone or SAGE‐217).mp. 1130

5 Allopregnanolone.mp. 2322

6 ((neurosteroid* or neuro* steroid* or neuroactive steroid* or positive allosteric modulat* or PAM?) and ((GABA? or gamma aminobutyric acid) and receptor*)).mp. 3269

7 exp 4 aminobutyric acid A receptor stimulating agent/ 16976

8 *GABAergic receptor affecting agent/ 303

9 or/1‐8 21427

10 postnatal depression/ 5211

11 ((postpartum* or post partum* or postnatal* or post natal* or perinatal* or peri natal* or puerp* or intrapartum* or intra partum* or antepartum* or ante partum*) adj3 (depress* or dysthymi* or adjustment disorder* or mood disorder* or affective disorder* or affective symptom*)).ti,ab,kw. 13209

12 (10 or 11) 15056

13 (9 and 12) 287

14 ((animal or nonhuman) not human).de. 6074097

15 (13 not 14) 273

16 (2021* or 2022*).yr,dp,dc. 2816573

17 (15 and 16) 77

18 limit 17 to yr="2021 ‐Current" 57

19 (17 or 18) 77

***************************************

**Ovid MEDLINE(R) ALL** <1946 to March 10, 2022>

1 (Brexanolone or Zulresso or SAGE‐547 or Ganaxolone or CCD‐1042 or Zuranolone or SAGE‐217).mp. 252

2 Allopregnanolone.mp. 1729

3 ((neurosteroid* or neuro* steroid* or neuroactive steroid* or positive allosteric modulat* or PAM?) and ((GABA? or gamma aminobutyric acid) and receptor*)).mp. 2557

4 1 or 2 or 3 3620

5 ((postpartum* or "post partum*" or postnatal* or "post natal*" or perinatal* or "peri natal*" or puerp* or intrapartum* or "intra partum*" or antepartum* or "ante partum*") and (depress* or dysthymi* or "adjustment disorder*" or "mood disorder*" or "affective disorder*" or "affective symptom*")).mp. 19165

6 (4 and 5) 151

7 exp animals/ not humans.sh. 4969949

8 (6 not 7) 131

9 (2021* or 2022*).yr,dp,dt,ep,ez. 2128800

10 (8 and 9) 36

11 limit 8 to yr="2021 ‐Current" 34

12 (10 or 11) 36

***************************************

**Ovid APA PsycInfo** <Inception to March Week 1 2022>

1 (Brexanolone or Zulresso or SAGE‐547 or Ganaxolone or CCD‐1042 or Zuranolone or SAGE‐217).mp. 86

2 Allopregnanolone.mp. 757

3 ((neurosteroid* or neuro* steroid* or neuroactive steroid* or positive allosteric modulat* or PAM?) and ((GABA? or gamma aminobutyric acid) and receptor*)).mp. 950

4 2 or 3 1400

5 ((postpartum* or "post partum*" or postnatal* or "post natal*" or perinatal* or "peri natal*" or puerp* or intrapartum* or "intra partum*" or antepartum* or "ante partum*") and (depress* or dysthymi* or "adjustment disorder*" or "mood disorder*" or "affective disorder*" or "affective symptom*")).mp. 13792

6 (4 and 5) 76

7 (1 or 6) 132

8 (2021* or 2022*).yr,an. 179967

9 (7 and 8) 26

***************************************

**Cochrane Central Register of Controlled Trials (CENTRAL)** (The Cochrane Library, Issue 3 of 12, 2022) (Searched 11 March 2022)

#1 (Brexanolone or Zulresso or SAGE‐547 or Ganaxolone or CCD‐1042 or Zuranolone or SAGE‐217) 148

#2 Allopregnanolone 152

#3 ((neurosteroid* or "neuro* steroid*" or "neuroactive steroid*" or "positive allosteric modulat*" or PAM or PAMs) and ((GABA* or "gamma aminobutyric acid") and receptor*)) 94

#4 (#2 or #3) 205

#5 ((postpartum* or "post partum*" or postnatal* or "post natal*" or perinatal* or "peri natal*" or puerp* or intrapartum* or "intra partum*" or antepartum* or "ante partum*") and (depress* or dysthymi* or "adjustment disorder*" or "mood disorder*" or "affective disorder*" or "affective symptom*")) 3742

#6 (#4 and #5) 44

#7 (#1 or #6) 137 Trials

Limited 1‐May‐2021 to 11‐Mar‐2022 n=15

***************************

**ClinicalTrials.gov** 11 March 2022

Brexanolone OR Zulresso OR SAGE‐547 (manually screened for PND) (1 new study)

Ganaxolone OR CCD‐1042 (screened for PND) (0 new studies since date of last search)

Zuranolone OR SAGE‐217 (screened for PND) (0 new studies since date of last search)

**WHO‐ICTRP**https://trialsearch.who.int (all years to date)

Brexanolone OR Zulresso OR SAGE‐547 (manually screened for PND) (7 studies)

Ganaxolone OR CCD‐1042 (screened for PND) (2 studies)

Zuranolone OR SAGE‐217 (screened for PND) (2 studies)

*************************************************************************

### Appendix 4. Additional reports of studies

**Table:**

**Study**	**Additional conference record**
**Deligiannidis 2021**	P.307 Effect of zuranolone on depression and anxiety outcomes in postpartum depression in a randomized, placebo‐controlled trial Mittal A; Deligiannidis KM; Huang MY; Suthoff E; Acaster S; Fridman M, et al. European Neuropsychopharmacology 2020;40 (Supplement 1):S177 2020 DOI: 10.1016/j.euroneuro.2020.09.231
**Deligiannidis 2021**	Effect of SAGE‐217 on anxiety outcomes in postpartum depression in a randomized, placebo‐controlled trial Mittal A; Deligiannidis K; Huang MY; Suthoff E; Acaster S; Fridman M, et al Biological Psychiatry 2020;87 (Supplement 9):S278‐9 2020 DOI: 10.1016/j.biopsych.2020.02.719
**Deligiannidis 2021**	Evaluation of insomnia symptoms in a doubleblind, randomized, placebo‐controlled phase 3 trial of sage‐217 in postpartum depression Mittal A; Deligiannidis K; Huang M; Suthoff E; Acaster S; Fridman M, et al. Sleep 2020;43 (Supplement 1):A204‐5 2020 DOI: 10.1093/sleep/zsaa056.532
**Deligiannidis 2021**	P.308 A double‐blind, randomized, placebo‐controlled phase 3 study of sage‐217 in postpartum depression: improvements in unidimensional measures of depression and anxiety Lasser R; Deligiannidis K; Gunduz‐Bruce H; Silber C; Sankoh A; Li S, et al. European Neuropsychopharmacology 2019;29 (Supplement 6):S219‐20 2019 DOI: 10.1016/j.euroneuro.2019.09.328
**Deligiannidis 2021**	A phase 3, double‐blind, placebo‐controlled trial of SAGE‐217 in postpartum depression: assessment of depressive symptoms across multiple measures Junker H. Pharmacopsychiatry 2020;53 (2):94 2020 DOI: 10.1055/s‐0039‐3403036
**Deligiannidis 2021**	934: Phase 3, randomized, placebo‐controlled trial of SAGE‐217 in postpartum depression: association between HAM‐D and PHQ‐9 Huang MY; Suthoff E; Deligiannidis K; Lasser R; Gunduz‐Bruce H; Silber C, et al. American Journal of Obstetrics and Gynecology 2020;222 (1 Supplement):S578 2020 DOI: 10.1016/j.ajog.2019.11.945
**Deligiannidis 2021**	SAGE‐217 in postpartum depression (PPD): number Nneeded to treat (NNT) from a phase 3, randomized, placebo‐controlled trial Huang MY; Deligiannidis K; Suthoff E; Mittal A; Werneburg B; Acaster S, et al. Biological Psychiatry 2020;87 (9 Supplement):S334‐5 2020 DOI: 10.1016/j.biopsych.2020.02.859
**Deligiannidis 2021**	Clinical global impression scores and number needed to treat outcomes in patients with postpartum depression treated with the oral neuroactive steroid zuranolone Deligiannidis K; Werneburg B; Huang MY; Suthoff E; Lasser R; Gunduz‐Bruce H. et al Neuropsychopharmacology 2020;45:323‐4 2020 DOI: 10.1038/s41386‐020‐00892‐5
**Deligiannidis 2021**	Evaluation of insomnia symptoms in a double‐blind, randomized, placebo‐controlled phase 3 trial of Zuranolone in postpartum depression Deligiannidis K; Huang MY; Suthoff E; Acaster S; Fridman M; Gunduz‐Bruce H, et al. Biological Psychiatry 2021;89 (Supplement 9): S91 2021 DOI: 10.1016/j.biopsych.2021.02.239
**Deligiannidis 2021**	Rapid and sustained improvement in concurrent symptoms of depression and anxiety in a post hoc analysis of Zuranolone treatment in postpartum depression Deligiannidis K; Huang MY; Acaster S; Fridman M; Gunduz‐Bruce H; Lasser R. et al. Biological Psychiatry 2021;89 (Supplement 9):S157 2021 DOI: 10.1016/j.biopsych.2021.02.404
**Deligiannidis 2021**	Evaluation of depression and anxiety in a phase 3, double‐blind, placebo‐controlled trial of the neuroactive steroid GABAA receptor positive allosteric modulator SAGE‐217 in postpartum depression Deligiannidis K; Lasser R; Gunduz‐Bruce H; Silber C; Sankoh A; Li S.et al. Neuropsychopharmacology 2019;44 (Supplement 1):426‐7 2019 DOI: 10.1038/s41386‐019‐0547‐9
**Deligiannidis 2021**	Health‐related quality of life in a phase 3, randomized, placebo‐controlled trial of the neuroactive steroid GABAA receptor positive allosteric modulator SAGE‐217 in postpartum depression Deligiannidis K; Huang MY; Suthoff E; Lasser R; Gunduz‐Bruce H; Silber C. et al. Neuropsychopharmacology 2019;44 (Supplement 1):299‐300 2019 DOI: 10.1038/s41386‐019‐0546‐x
**Kanes 2017b**	SAGE‐547 for the treatment of severe postpartum depression Kanes S; Colquhoun H; Gunduz‐Bruce H; Doherty J; Jonas J; Rubinow D. et al. Neuropsychopharmacology 2016;41 (Supplement 1):S165‐6 2016 DOI: 10.1038/npp.2016.240
**Meltzer‐Brody 2018 Study 1**	Phase 3 study evaluating brexanolone, a gabaa receptor modulator, in severe postpartum depression Meltzer‐Brody S; Kanes S; Riesenberg R; Rubinow D; Maximos B; Colquhoun H. Obstetrics and Gynecology 2018;131 (Supplement 1):27S 2018

## Data and analyses

**Comparison 1:** Intravenous neurosteroid GABA_A_ positive allosteric modulators versus placebo

Outcome or subgroup title	No. of studies	No. of participants	Statistical method	Effect size
1.1 Depression response (early phase)	4		Risk Ratio (M‐H, Random, 95% CI)	Subtotals only
1.1.1 2 hours post‐infusion	3	267	Risk Ratio (M‐H, Random, 95% CI)	1.03 [0.37, 2.88]
1.1.2 4 hours post‐infusion	3	267	Risk Ratio (M‐H, Random, 95% CI)	1.13 [0.62, 2.06]
1.1.3 8 hours post‐infusion	3	267	Risk Ratio (M‐H, Random, 95% CI)	1.11 [0.70, 1.74]
1.1.4 12 hours post‐infusion	4	325	Risk Ratio (M‐H, Random, 95% CI)	0.88 [0.60, 1.29]
1.1.5 24 hours post‐infusion	4	325	Risk Ratio (M‐H, Random, 95% CI)	1.17 [0.81, 1.70]
1.1.6 36 hours post‐infusion	3	267	Risk Ratio (M‐H, Random, 95% CI)	1.32 [0.99, 1.77]
1.1.7 48 hours post‐infusion	4	325	Risk Ratio (M‐H, Random, 95% CI)	1.17 [0.91, 1.50]
1.1.8 60 hours post‐infusion	4	325	Risk Ratio (M‐H, Random, 95% CI)	1.27 [1.05, 1.54]
1.1.9 72 hours post‐infusion	4	325	Risk Ratio (M‐H, Random, 95% CI)	1.25 [1.03, 1.51]
1.1.10 7 days post‐infusion	3	267	Risk Ratio (M‐H, Random, 95% CI)	1.34 [0.90, 1.99]
1.1.11 30 days post‐infusion	3	267	Risk Ratio (M‐H, Random, 95% CI)	1.24 [0.74, 2.06]
1.2 Depression remission (early phase)	4		Risk Ratio (M‐H, Random, 95% CI)	Subtotals only
1.2.1 2 hours post‐infusion	2	129	Risk Ratio (M‐H, Random, 95% CI)	1.00 [0.06, 15.58]
1.2.2 4 hours post‐infusion	2	129	Risk Ratio (M‐H, Random, 95% CI)	0.80 [0.16, 3.93]
1.2.3 8 hours post‐infusion	3	267	Risk Ratio (M‐H, Random, 95% CI)	1.23 [0.18, 8.48]
1.2.4 12 hours post‐infusion	4	325	Risk Ratio (M‐H, Random, 95% CI)	1.52 [0.47, 4.91]
1.2.5 24 hours post‐infusion	4	325	Risk Ratio (M‐H, Random, 95% CI)	1.60 [0.91, 2.82]
1.2.6 36 hours post‐infusion	3	267	Risk Ratio (M‐H, Random, 95% CI)	1.49 [0.92, 2.42]
1.2.7 48 hours post‐infusion	4	325	Risk Ratio (M‐H, Random, 95% CI)	1.60 [1.06, 2.40]
1.2.8 60 hours post‐infusion	4	325	Risk Ratio (M‐H, Random, 95% CI)	1.68 [1.01, 2.80]
1.2.9 72 hours post‐infusion	4	325	Risk Ratio (M‐H, Random, 95% CI)	1.65 [1.19, 2.28]
1.2.10 7 days post‐infusion	3	267	Risk Ratio (M‐H, Random, 95% CI)	1.45 [0.59, 3.56]
1.2.11 30 days post‐infusion	3	267	Risk Ratio (M‐H, Random, 95% CI)	1.18 [0.59, 2.38]
1.3 Adverse events (mother)	4		Risk Ratio (M‐H, Random, 95% CI)	Subtotals only
1.3.1 Any adverse event	4	325	Risk Ratio (M‐H, Random, 95% CI)	1.02 [0.71, 1.48]
1.3.2 Severe adverse event	3	267	Risk Ratio (M‐H, Random, 95% CI)	1.17 [0.23, 5.89]
1.3.3 Serious adverse event	4	325	Risk Ratio (M‐H, Random, 95% CI)	2.13 [0.23, 20.21]
1.4 Depression severity according to HAMD (early phase)	4		Mean Difference (IV, Random, 95% CI)	Subtotals only
1.4.1 2 hours post‐infusion	3		Mean Difference (IV, Random, 95% CI)	‐0.29 [‐1.22, 0.64]
1.4.2 4 hours post‐infusion	3		Mean Difference (IV, Random, 95% CI)	‐1.12 [‐2.30, 0.06]
1.4.3 8 hours post‐infusion	3		Mean Difference (IV, Random, 95% CI)	‐1.27 [‐2.56, 0.03]
1.4.4 12 hours post‐infusion	4		Mean Difference (IV, Random, 95% CI)	‐0.45 [‐1.88, 0.97]
1.4.5 24 hours post‐infusion	4		Mean Difference (IV, Random, 95% CI)	‐2.96 [‐5.26, ‐0.66]
1.4.6 36 hours post‐infusion	3		Mean Difference (IV, Random, 95% CI)	‐3.70 [‐6.62, ‐0.79]
1.4.7 48 hours post‐infusion	4		Mean Difference (IV, Random, 95% CI)	‐3.77 [‐5.91, ‐1.64]
1.4.8 60 hours post‐infusion	4		Mean Difference (IV, Random, 95% CI)	‐3.75 [‐6.13, ‐1.37]
1.4.9 72 hours post‐infusion	4		Mean Difference (IV, Random, 95% CI)	‐3.64 [‐6.00, ‐1.28]
1.4.10 7 days post‐infusion	3		Mean Difference (IV, Random, 95% CI)	‐4.11 [‐7.08, ‐1.14]
1.4.11 30 days post‐infusion	3		Mean Difference (IV, Random, 95% CI)	‐4.22 [‐8.46, 0.02]
1.5 Treatment acceptability (measured by dropouts)	3	267	Risk Ratio (M‐H, Random, 95% CI)	2.77 [1.22, 6.26]

**Comparison 2:** Oral zuranolone versus placebo

Outcome or subgroup title	No. of studies	No. of participants	Statistical method	Effect size
2.1 Depression response (early phase)	2		Risk Ratio (M‐H, Random, 95% CI)	Subtotals only
2.1.1 Day 3	2	349	Risk Ratio (M‐H, Random, 95% CI)	1.70 [1.17, 2.47]
2.1.2 Day 8	2	349	Risk Ratio (M‐H, Random, 95% CI)	1.64 [1.24, 2.18]
2.1.3 Day 15	2	349	Risk Ratio (M‐H, Random, 95% CI)	1.50 [1.21, 1.86]
2.1.4 Day 21	2	349	Risk Ratio (M‐H, Random, 95% CI)	1.33 [1.09, 1.63]
2.1.5 Day 28	1	196	Risk Ratio (M‐H, Random, 95% CI)	1.37 [0.98, 1.91]
2.2 Depression response (acute phase)	2	349	Risk Ratio (M‐H, Random, 95% CI)	1.26 [1.03, 1.55]
2.3 Depression remission (early phase)	2		Risk Ratio (M‐H, Random, 95% CI)	Subtotals only
2.3.1 Day 3	2	349	Risk Ratio (M‐H, Random, 95% CI)	2.37 [1.11, 5.05]
2.3.2 Day 8	2	349	Risk Ratio (M‐H, Random, 95% CI)	1.63 [1.04, 2.55]
2.3.3 Day 15	2	349	Risk Ratio (M‐H, Random, 95% CI)	1.81 [1.24, 2.63]
2.3.4 Day 21	2	349	Risk Ratio (M‐H, Random, 95% CI)	1.46 [1.03, 2.08]
2.3.5 Day 28	1	196	Risk Ratio (M‐H, Random, 95% CI)	1.30 [0.78, 2.17]
2.4 Depression remission (acute phase)	2	349	Risk Ratio (M‐H, Random, 95% CI)	1.65 [1.22, 2.22]
2.5 Adverse events (mother)	2		Risk Ratio (M‐H, Random, 95% CI)	Subtotals only
2.5.1 Any adverse events	2	349	Risk Ratio (M‐H, Random, 95% CI)	1.24 [1.03, 1.48]
2.5.2 Severe adverse events	2	349	Risk Ratio (M‐H, Random, 95% CI)	1.42 [0.39, 5.14]
2.5.3 Serious adverse events	2	349	Risk Ratio (M‐H, Random, 95% CI)	2.06 [0.27, 15.77]
2.5.4 Deaths	2	349	Risk Ratio (M‐H, Random, 95% CI)	Not estimable
2.6 Depression severity (early phase)	2		Mean Difference (IV, Random, 95% CI)	Subtotals only
2.6.1 Day 3	2		Mean Difference (IV, Random, 95% CI)	‐3.10 [‐4.62, ‐1.59]
2.6.2 Day 8	2		Mean Difference (IV, Random, 95% CI)	‐3.58 [‐5.20, ‐1.96]
2.6.3 Day 15	2		Mean Difference (IV, Random, 95% CI)	‐4.08 [‐5.83, ‐2.34]
2.6.4 Day 21	2		Mean Difference (IV, Random, 95% CI)	‐2.75 [‐4.58, ‐0.93]
2.6.5 Day 28	1		Mean Difference (IV, Random, 95% CI)	‐2.90 [‐5.35, ‐0.45]
2.7 Depression severity (acute phase)	2		Mean Difference (IV, Random, 95% CI)	‐3.79 [‐5.60, ‐1.97]
2.8 Treatment acceptability	2	349	Risk Ratio (M‐H, Random, 95% CI)	0.95 [0.50, 1.81]
2.9 Barkin Index of Maternal Functioning (early phase)	1		Mean Difference (IV, Random, 95% CI)	Subtotals only
2.9.1 Day 3	1		Mean Difference (IV, Random, 95% CI)	2.20 [‐1.19, 5.59]
2.9.2 Day 8	1		Mean Difference (IV, Random, 95% CI)	1.10 [‐3.19, 5.39]
2.9.3 Day 15	1		Mean Difference (IV, Random, 95% CI)	4.70 [‐0.75, 10.15]
2.9.4 Day 21	1		Mean Difference (IV, Random, 95% CI)	3.60 [‐1.89, 9.09]
2.10 Barkin Index of Maternal Functioning (acute phase)	1		Mean Difference (IV, Random, 95% CI)	Subtotals only

**Comparison 3:** Subgroup analysis by individual drug (Brexanolone only)

Outcome or subgroup title	No. of studies	No. of participants	Statistical method	Effect size
3.1 Depression response (early phase)	3		Risk Ratio (M‐H, Random, 95% CI)	Subtotals only
3.1.1 2 hours post‐infusion	3	267	Risk Ratio (M‐H, Random, 95% CI)	1.03 [0.37, 2.88]
3.1.2 4 hours post‐infusion	3	267	Risk Ratio (M‐H, Random, 95% CI)	1.13 [0.62, 2.06]
3.1.3 8 hours post‐infusion	3	267	Risk Ratio (M‐H, Random, 95% CI)	1.11 [0.70, 1.74]
3.1.4 12 hours post‐infusion	3	267	Risk Ratio (M‐H, Random, 95% CI)	0.89 [0.61, 1.32]
3.1.5 24 hours post‐infusion	3	267	Risk Ratio (M‐H, Random, 95% CI)	1.24 [0.90, 1.72]
3.1.6 36 hours post‐infusion	3	267	Risk Ratio (M‐H, Random, 95% CI)	1.32 [0.99, 1.77]
3.1.7 48 hours post‐infusion	3	267	Risk Ratio (M‐H, Random, 95% CI)	1.23 [0.90, 1.69]
3.1.8 60 hours post‐infusion	3	267	Risk Ratio (M‐H, Random, 95% CI)	1.29 [1.05, 1.59]
3.1.9 72 hours post‐infusion	3	267	Risk Ratio (M‐H, Random, 95% CI)	1.30 [0.97, 1.73]
3.1.10 7 days post‐infusion	3	267	Risk Ratio (M‐H, Random, 95% CI)	1.34 [0.90, 1.99]
3.1.11 30 days post‐infusion	3	267	Risk Ratio (M‐H, Random, 95% CI)	1.24 [0.74, 2.06]
3.2 Depression remission (early phase)	3		Risk Ratio (M‐H, Random, 95% CI)	Subtotals only
3.2.1 2 hours post‐infusion	2	129	Risk Ratio (M‐H, Random, 95% CI)	1.00 [0.06, 15.58]
3.2.2 4 hours post‐infusion	2	129	Risk Ratio (M‐H, Random, 95% CI)	0.80 [0.16, 3.93]
3.2.3 8 hours post‐infusion	3	267	Risk Ratio (M‐H, Random, 95% CI)	1.23 [0.18, 8.48]
3.2.4 12 hours post‐infusion	3	267	Risk Ratio (M‐H, Random, 95% CI)	1.52 [0.47, 4.91]
3.2.5 24 hours post‐infusion	3	267	Risk Ratio (M‐H, Random, 95% CI)	1.85 [0.85, 4.06]
3.2.6 36 hours post‐infusion	3	267	Risk Ratio (M‐H, Random, 95% CI)	1.49 [0.92, 2.42]
3.2.7 48 hours post‐infusion	3	267	Risk Ratio (M‐H, Random, 95% CI)	1.73 [1.12, 2.68]
3.2.8 60 hours post‐infusion	3	267	Risk Ratio (M‐H, Random, 95% CI)	1.98 [1.09, 3.61]
3.2.9 72 hours post‐infusion	3	267	Risk Ratio (M‐H, Random, 95% CI)	1.81 [1.26, 2.60]
3.2.10 7 days post‐infusion	3	267	Risk Ratio (M‐H, Random, 95% CI)	1.45 [0.59, 3.56]
3.2.11 30 days post‐infusion	3	267	Risk Ratio (M‐H, Random, 95% CI)	1.18 [0.59, 2.38]
3.3 Adverse events (mother)	3		Risk Ratio (M‐H, Random, 95% CI)	Subtotals only
3.3.1 Any adverse event	3	267	Risk Ratio (M‐H, Random, 95% CI)	0.93 [0.71, 1.21]
3.3.2 Severe adverse event	3	267	Risk Ratio (M‐H, Random, 95% CI)	1.17 [0.23, 5.89]
3.3.3 Serious adverse event	3	267	Risk Ratio (M‐H, Random, 95% CI)	2.13 [0.23, 20.21]
3.4 Depression severity according to HAMD (early phase)	3		Mean Difference (IV, Random, 95% CI)	Subtotals only
3.4.1 2 hours post‐infusion	3		Mean Difference (IV, Random, 95% CI)	‐0.29 [‐1.22, 0.64]
3.4.2 4 hours post‐infusion	3		Mean Difference (IV, Random, 95% CI)	‐1.12 [‐2.30, 0.06]
3.4.3 8 hours post‐infusion	3		Mean Difference (IV, Random, 95% CI)	‐1.27 [‐2.56, 0.03]
3.4.4 12 hours post‐infusion	3		Mean Difference (IV, Random, 95% CI)	‐0.89 [‐2.36, 0.58]
3.4.5 24 hours post‐infusion	3		Mean Difference (IV, Random, 95% CI)	‐3.59 [‐6.29, ‐0.90]
3.4.6 36 hours post‐infusion	3		Mean Difference (IV, Random, 95% CI)	‐3.70 [‐6.62, ‐0.79]
3.4.7 48 hours post‐infusion	3		Mean Difference (IV, Random, 95% CI)	‐4.18 [‐6.82, ‐1.54]
3.4.8 60 hours post‐infusion	3		Mean Difference (IV, Random, 95% CI)	‐4.45 [‐6.99, ‐1.91]
3.4.9 72 hours post‐infusion	3		Mean Difference (IV, Random, 95% CI)	‐4.27 [‐6.62, ‐1.92]
3.4.10 7 days post‐infusion	3		Mean Difference (IV, Random, 95% CI)	‐4.11 [‐7.08, ‐1.14]
3.4.11 30 days post‐infusion	3		Mean Difference (IV, Random, 95% CI)	‐4.22 [‐8.46, 0.02]
3.5 Treatment acceptability (measured by dropouts)	3	267	Risk Ratio (M‐H, Random, 95% CI)	2.77 [1.22, 6.26]

## Characteristics of studies

### Characteristics of included studies [ordered by study ID]

**Table:** Deligiannidis 2021

***Study characteristics***		
Methods	**Study design:** RCT **Location:** USA **Setting:** outpatient clinics **No. of centres:** 33 approved, 27 recruited **Dates of study**: January 2017 to December 2018 **Total duration of study:** 24 months **Recruitment**: not reported **Randomisation method:** computer‐generated randomisation program	
Participants	**Inclusion criteria**: adult ambulatory female patients, aged 18 to 45 years old, six months or less postpartum, with a major depressive episode without psychosis (diagnosed by SCID for DSM‐5 Axis I Disorders for clinical trials) that began no earlier than the 3rd trimester and no later than the first 4 weeks following delivery, were enrolled. A baseline HAMD‐17 score of 26 or higher was required. Patients taking psychotropic medications used to treat depressive symptoms were required to have been taking a stable dose for more than 30 days prior to day 1 and delay the start/alteration of psychotropic treatment regimens until after the treatment period and day 15 assessments were completed. Patients must have ceased lactating at screening or agreed to cease breastfeeding from just prior to receiving the study drug until seven days after the last dose. **Exclusion criteria**: active psychosis; attempted suicide associated with current episode of postpartum depression; medical history of seizures or medical history of bipolar disorder, schizophrenia, and/or schizoaffective disorder. **Number recruited**: 153: zuranolone n = 77; placebo n = 76 **Number dropped out**: 11: zuranolone n = 4; placebo n = 7 **Number analysed**: 150: zuranolone n = 76; placebo n = 74 **Age, mean (SD) years**: zuranolone 29.3 (5.4); placebo 27.4 (5.3) **Severity of PND, baseline HAMD‐17 mean (SD) score**: zuranolone 28.4 (2); placebo 28.8 (2) **Onset of PND:** zuranolone 42% third trimester, 58% < 4 weeks after delivery; placebo 42% third trimester, 58% < 4 weeks after delivery **Previous PND:** not reported **Family history of PND**: zuranolone 13%, placebo 14% **Physical health comorbidities**: not reported **Mental health comorbidities**: not reported **Use of antidepressant medication at baseline:** zuranolone 21% placebo 18%	
Interventions	**Zuranolone:** 30 mg once daily (dose could be reduced to 20 mg if participants were unable to tolerate 30 mg per day) **Placebo**: matching capsules **Duration**: 2 weeks	
Outcomes	**Primary outcome:** change from baseline in total HAMD‐17 score (day 15) **Secondary outcome:** change from baseline in total HAMD‐17 score at other time points (days 3, 8, 21, and 45), MADRS, HAM‐A, HAMD‐17 response, HAMD‐17 remission, CGI‐I, change in baseline from BIMF, adverse events **Time points:** days 3, 8, 21 and 45	
Notes	Funded by Sage Therapeutics, Inc.	
***Risk of bias***		
**Bias**	**Authors' judgement**	**Support for judgement**
Random sequence generation (selection bias)	Low risk	Computer‐generated randomisation schedule produced by an independent statistician
Allocation concealment (selection bias)	Low risk	"Randomized by interactive response technology implementation"
Blinding of participants and personnel (performance bias) All outcomes	Low risk	Study site–designated pharmacy staff, responsible for dispensing the study drug, were the only study personnel unblinded to the randomisation scheme. All other site personnel were blinded to treatment assignments during the study.
Blinding of outcome assessment (detection bias) All outcomes	Low risk	Study site–designated pharmacy staff, responsible for dispensing the study drug, were the only study personnel unblinded to the randomisation scheme. All other site personnel were blinded to treatment assignments during the study.
Incomplete outcome data (attrition bias) All outcomes	Low risk	Reasons for participants discontinuing the study are clearly explained. Discontinuation rates appear balanced across groups.
Selective reporting (reporting bias)	Low risk	All prespecified outcomes were reported.
Other bias	Unclear risk	The trial was funded by the drug manufacturer, who appears to have had a considerable role in the design and conduct of the trial. Many of the authors also declared financial conflicts of interest as employees or stock owners of the drug manufacturer. It is unclear if these circumstances may have biased the trial findings.

**Table:** Deligiannidis 2023

***Study characteristics***		
Methods	**Study design:** randomised, double‐blind, placebo‐controlled, phase 3 trial **Location**: USA **Setting**: not reported **Number of centres**: 82 **Dates of study**: June 2020 to April 2022 **Total duration of study:** 2 years **Recruitment**: not reported **Randomisation method**: not reported	
Participants	**Inclusion criteria**: 18‐ to 24‐year‐old women with a baseline score ≥ 26 on HAM‐D 17 who had a major depressive episode with onset during the third trimester of pregnancy or ≤ 4 weeks postpartum or were ≤ 12 weeks postpartum **Exclusion criteria**: history of bipolar disorder, schizophrenia, and/or schizoaffective disorder, psychotic disorders, medical history of nonfebrile seizures, history of sleep apnoea, attempted suicide, or risk of suicide in the current episode of PPD. **Number recruited**: 196: zuranolone n = 98; placebo n = 98 **Number dropped out**: 26: zuranolone n = 14; placebo n = 12 **Number analysed**: 195: zuranolone n = 98; placebo n = 97 **Age, mean (SD) years**: zuranolone 30 (SD 5.9) years, placebo 31 (SD 6.0) years **Severity of PND, baseline HAMD‐17 mean (SD) score**: zuranolone 28.6 (2.5), placebo 28.8 (2.3) **Onset of PND:** zuranolone 34.7% third trimester, 65.3% ≤ 4 weeks postpartum; placebo 31.6% third trimester, 68.4% ≤ 4 weeks postpartum **Previous PND:** zuranolone 17.3%, placebo 11.2% **Family history of PND**: not reported **Physical health comorbidities**: not reported **Mental health comorbidities**: zuranolone mean (SD) HAM‐A 24.4 (6.0), placebo 24.7 (6.0) **Use of antidepressant medication at baseline:** zuranolone 15.3%, placebo 15.3%	
Interventions	**Zuranolone:** 50 mg once daily in the evening. Patients unable to tolerate zuranolone at 50 mg/day were permitted a reduction to 40 mg/day for the remainder of the treatment course **Placebo**: matching capsules **Duration**: 2 weeks	
Outcomes	**Primary outcome:** change from baseline in total HAMD‐17 score (day 15) **Secondary outcome:** change from baseline in total HAMD‐17 score at other time points (days 3, 28 and 45), CGI‐S (day 15), HAM‐D response (days 15 and 45), HAM‐D remission (days 15 and 45), CGI‐I response (day 15), change from baseline in total HAM‐A (day 15), change from baseline in total MADRS score (day 15), change from baseline in HAM‐D subscale (day 15), change from baseline in total EPDS score (days 3, 8, 15, 21, 28 and 45), change from baseline in PHQ‐9 score (days 3, 8, 15, 21, 28 and 45), treatment‐emergent adverse events. Suicide ideation behaviour was evaluated using the Columbia–Suicide Severity Rating Scale (C‐SSRS) and potential withdrawal symptoms were monitored with the 20‐item Physician Withdrawal Checklist (PWC‐20) **Time points:** days 3, 15, 28 and 45	
Notes	Funded by Sage Therapeutics and Biogen	
***Risk of bias***		
**Bias**	**Authors' judgement**	**Support for judgement**
Random sequence generation (selection bias)	Unclear risk	Not reported
Allocation concealment (selection bias)	Unclear risk	Not reported
Blinding of participants and personnel (performance bias) All outcomes	Low risk	Patients, clinicians, and study personnel were blinded to treatment allocation during the study.
Blinding of outcome assessment (detection bias) All outcomes	Low risk	Patients, clinicians, and study personnel were blinded to treatment allocation during the study.
Incomplete outcome data (attrition bias) All outcomes	Unclear risk	The study followed a modified intention‐to‐treat approach where they analysed participants in the groups they were randomised to, but didn't include missing data due to loss to follow‐up or where participants did not have at least one dose of the study drug (did not start the treatment). Authors only excluded a small number of participants from the analysis due to additional criteria (at least one dose of study drug and baseline measurement) and it was balanced between groups. However, a higher number of participants in the zuranolone group (21.4% at day 28 compared to 12.4% in the placebo group) were lost to follow‐up or discontinued treatment, so this could have introduced bias.
Selective reporting (reporting bias)	Low risk	Study protocol is available and all prespecified outcomes are reported.
Other bias	Unclear risk	The trial was funded by the drug manufacturer, who appears to have had a considerable role in the design and conduct of the trial. Many of the authors also declared financial conflicts of interest as employees or stock owners of the drug manufacturer. It is unclear if these circumstances may have biased the trial findings.

**Table:** Kanes 2017

***Study characteristics***		
Methods	**Study design:** RCT **Location:** USA **Setting:** hospital **No. of centres:** 11 approved, 4 recruited **Dates of study**: December 2015 to May 2016 **Total duration of study:** 6 months **Recruitment**:self‐referred or physician‐referred **Randomisation method**: computer‐generated randomisation program	
Participants	**Inclusion criteria**: "ambulatory female aged between 18 and 45 years; good physical health and no clinically significant findings as determined by the investigator on physical examination, 12‐lead ECG, or clinical laboratory tests; agreed to adhere to the study requirements; had a negative pregnancy test at screening and day 1 before the start of study drug infusion; had a major depressive episode that began no earlier than the 3rd trimester and no later than the first 4 weeks following delivery as diagnosed by SCID for DSM‐V Axis I Disorders; had a HAMD‐17 total score of 26 or higher at screening and day one (before randomisation); was within six months post partum at the time of enrolment; and had no detectable hepatitis B surface antigen, anti‐hepatitis C virus, or HIV antibody at screening. Patients either must have ceased lactating at screening or if still lactating at screening, must have already fully and permanently weaned their infant(s) from breast milk, or if still actively breastfeeding at screening, must have agreed to cease giving breast milk to their infant(s) before receiving study drug. Signed informed consent form before any study‐specific procedures were performed." **Exclusion criteria**: active psychosis; attempted suicide associated with an index case of postpartum depression; history of seizures, bipolar disorder, schizophrenia, or schizoaffective disorder; and history of alcoholism or drug addiction (including benzodiazepines) in the 12 months before screening. **Number recruited**: n = 21 **Number dropped out**: none **Number analysed**: 21: brexanolone n = 10, placebo n = 11 **Age, mean (SD) years**: brexanolone 27.4 (5.3); placebo 28.8 (4.6) **Severity of HAM‐D, mean (range) score**: brexanolone 28.1 (27 to 30); placebo 28.8 (26 to 32) **Duration of PND**: not reported **Previous PND**: brexanolone n = 70%, placebo n = 36% **Family history of PND**: brexanolone n = 30%, placebo n = 27% **Physical health comorbidities**: not reported **Mental health comorbidities**: depression (non‐PPD): brexanolone n = 60%, placebo n = 55%; anxiety: brexanolone n = 20%, placebo n = 45%; other psychiatric disorder: brexanolone n = 10%, placebo n = 18% **Use of antidepressant medication at baseline:** brexanolone n = 30%, placebo n = 27%	
Interventions	**Brexanolone**: 30 μg/kg/h (0 to 4 hours); 60 μg/kg/h (4 to 24 hours); 90 μg/kg/h (24 to 52 hours); 60 μg/kg/h (52 to 56 hours); 30 μg/kg/h (56 to 60 hours) **Placebo**: matching infusion **Duration**: 60 hours	
Outcomes	**Primary outcome:** change from baseline in HAM‐D total score (60 hours) **Secondary outcome:** HAM‐D response, HAM‐D remission, MADRS total score, CGI‐I, HAM‐D Bech 6 sub‐scale, HAM‐D individual item scores, GAD‐7 total score, treatment‐emergent adverse events **Time points:** 60 hours, days 7 and 30	
Notes	Funded by Sage Therapeutics, Inc. ClinicalTrials.gov number NCT02614547 We corresponded by email with Sage Therapeutics Inc to request data for time points only presented graphically. We received no response from Sage and instead used PlotDigitizer to extract data from Figure 3 of Kanes 2017.	
***Risk of bias***		
**Bias**	**Authors' judgement**	**Support for judgement**
Random sequence generation (selection bias)	Low risk	Computer‐generated randomisation schedule produced by an independent statistician
Allocation concealment (selection bias)	Low risk	Only the trial pharmacist had access to the randomisation schedule.
Blinding of participants and personnel (performance bias) All outcomes	Low risk	Patients, clinicians, and study teams were masked to treatment allocation.
Blinding of outcome assessment (detection bias) All outcomes	Low risk	The study team was not given access to the study data until after the final participant had completed their final follow‐up visit.
Incomplete outcome data (attrition bias) All outcomes	Low risk	All participants completed the trial.
Selective reporting (reporting bias)	Low risk	All prespecified outcomes were reported.
Other bias	Unclear risk	The trial was funded by the drug manufacturer, who appears to have had a considerable role in the design and conduct of the trial. Many of the authors also declared financial conflicts of interest as employees or stock owners of the drug manufacturer. It is unclear if these circumstances may have biased the trial findings.

**Table:** Meltzer‐Brody 2018 Study 1

***Study characteristics***		
Methods	**Study design:** RCT **Location:** USA **Setting:** clinical research centres and specialised psychiatric units **No. of centres:** 30 **Dates of study**: August 2016 to October 2017 **Total duration of study:** 14 months **Recruitment**: self‐referrals, physician referrals, and radio‐based, television‐based, and web‐based methods **Randomisation method**: computer‐generated randomisation program	
Participants	**Inclusion criteria**: "ambulatory female participants aged 18 to 45 years, who provided a signed informed consent form before any procedures were done, were in good physical health with no clinically significant findings as determined by the principal investigator on physical examination, 12‐lead ECG, or clinical laboratory tests, had agreed to adhere to study requirements, and had stopped lactating at the time of screening or had temporarily ceased breastfeeding while receiving the study drug until four days after the end of infusion. Eligible participants also had to have a negative pregnancy test before initiation of study drug, had a major depressive episode with onset no earlier than the third trimester and no later than four weeks after delivery, as determined by SCID for DSM‐IV Axis I Disorders, had a qualifying HAM‐D total score (≥ 26 for study 1; 20 to 25 for study 2) before infusion, were 6 months post partum or less at screening, were willing to delay the start of new pharmacotherapeutic drug regimens from study drug infusion until completion of 72 hour assessments, and agreed to use an approved form of birth control during the study and for 30 days thereafter. Women with onset of post‐partum depression in the peripartum period were included on the basis of previous literature demonstrating postpartum depression development in this time period. Participants taking prescribed psychotropic medication at baseline had to be at a stable dose 14 days before screening until completion of the 72 hour assessments." **Exclusion criteria**: "individuals were excluded if they had renal failure requiring dialysis, fulminant hepatic failure, anaemia (baseline haemoglobin < 10 g/dL), known allergy to allopregnanolone or to progesterone, active psychosis (determined by investigator), medical history of schizophrenia, bipolar disorder, or schizoaffective disorder, had attempted suicide during the current episode of postpartum depression, or a history of alcohol or drug abuse in the previous 12 months (by self‐report or drug screening). Individuals were also excluded if they had been exposed to another investigational medication up to 30 days before screening, had previously participated in this study or other studies of brexanolone injection or had ECT up to 14 days before screening or had therapy planned within seven days after infusion." **Number recruited**: n = 138: brexanolone 60 μg n = 47, brexanolone 90 μg n = 45, placebo n = 46 **Number dropped out**: n = 25: brexanolone 60 μg n = 12, brexanolone 90 μg n = 9, placebo n = 4 **Number analysed**: brexanolone 60 μg n = 38, brexanolone 90 μg n = 41, placebo n = 43 **Age (mean) years**: brexanolone 60 μg: 27.3 (6.1), brexanolone 90 μg: 27.8 (6.0), placebo: 27 (6) **Severity of PND, mean HAM‐D (SD) score**: brexanolone 60 μg 29.1 (2.7), brexanolone 90 μg: 28.4 (2.5), placebo: 28.6 (2.5) **Onset of PND**: third trimester: brexanolone 60 μg n = 23%, brexanolone 90 μg n = 22%, placebo n = 30%; within 4 weeks of delivery: brexanolone 60 μg n = 74%, brexanolone 90 μg n = 78%, placebo n = 67% **Previous PND:** brexanonlone 60 μg n = 36%, brexanolone 90 μg n = 27%, placebo n = 35% **Family history of PND**: brexanonlone 60 μg n = 28%, brexanolone 90 μg n = 36%, placebo n = 20% **Physical health comorbidities**: not reported **Mental health comorbidities**: depression (non‐PPD): brexanolone 60 μg n = 40%, brexanolone 90 μg n = 47%, placebo n = 43%; anxiety: brexanolone 60 μg n = 43%, brexanolone 90 μg n = 47%, placebo n = 33%; premenstrual dysphoric disorder: brexanolone 60 μg n = 2%, brexanolone 90 μg n = 7%, placebo n = 0%; other psychiatric disorder: brexanolone 60 μg n = 11%; brexanolone 90 μg n = 4%; placebo n = 7% **Use of antidepressant medication at baseline:** brexanolone 60 μg n = 26%, brexanolone 90 μg n = 22%, placebo n = 26%	
Interventions	**Brexanolone 60 μg/kg/h**: single continuous infusion of study drug for 60 h according to the following schedule: 30 μg/kg per h (0 to 4 h); 60 μg/kg per h (4 to 24 h); 60 μg/kg per h (24 to 52 h); 60 μg/kg per h (52 to 56 h); 30 μg/kg per h (56 to 60 h) **Brexanolone 90 μg/kg/h:** single continuous infusion of study drug for 60 h according to the following schedule: 30 μg/kg per h (0 to 4 h); 60 μg/kg per h (4 to 24 h); 90 μg/kg per h (24 to 52 h); 60 μg/kg per h (52 to 56 h); 30 μg/kg per h (56 to 60 h) **Placebo**: matching infusion **Duration**: 60 hours	
Outcomes	**Primary outcome:** change from baseline in HAM‐D total score at 60 h post‐infusion **Secondary outcome:** mean HAM‐D total score, CGI response, change from baseline in MADRS, EPDS and HAM‐D sub‐scale, PHQ and GAD‐7 **Time points:** 0, 2, 4, 8, 12, 24, 36, 48, 60, and 72 h after infusion and follow‐up days 7 and 30	
Notes	Funded by Sage Therapeutics, Inc. We corresponded by email with Sage Therapeutics Inc. We were sent by Sage (on 28 September 2022) response and remission data for this study at day 30. We requested these data as they were only presented graphically in the published manuscript, and they were the most distal time point (Meltzer‐Brody 2018 Study 1). When we decided later to request data for the other time points, we received no response from Sage and instead used PlotDigitizer to extract data from Figures S1 and S2 of Meltzer‐Brody 2018 Study 1.	
***Risk of bias***		
**Bias**	**Authors' judgement**	**Support for judgement**
Random sequence generation (selection bias)	Low risk	Randomisation schedule provided by an independent third party
Allocation concealment (selection bias)	Low risk	Only the study pharmacist was aware of treatment allocation.
Blinding of participants and personnel (performance bias) All outcomes	Low risk	Participants and study personnel were masked to group allocation.
Blinding of outcome assessment (detection bias) All outcomes	Low risk	The study team did not have access to the trial database until the final enrolled participant had completed their final visit.
Incomplete outcome data (attrition bias) All outcomes	Low risk	Reasons for participants discontinuing the study are clearly explained. Discontinuation rates appear balanced across groups.
Selective reporting (reporting bias)	Low risk	All prespecified outcomes are reported.
Other bias	Unclear risk	The trial was funded by the drug manufacturer, who appears to have had a considerable role in the design and conduct of the trial. Many of the authors also declared financial conflicts of interest as employees or stock owners of the drug manufacturer. It is unclear if these circumstances may have biased the trial findings.

**Table:** Meltzer‐Brody 2018 Study 2

***Study characteristics***		
Methods	**Study design:** RCT **Location:** USA **Setting:** clinical research centres and specialised psychiatric units **No. of centres:** 30 **Dates of study**: July 2016 to October 2017 **Total duration of study:** 15 months **Recruitment**: self‐referrals, physician referrals, and radio‐based, television‐based, and web‐based methods **Randomisation method**: computer‐generated randomisation program	
Participants	**Inclusion criteria**: "ambulatory female participants aged 18 to 45 years, who provided a signed informed consent form before any procedures were done, were in good physical health with no clinically significant findings as determined by the principal investigator on physical examination, 12‐lead ECG, or clinical laboratory tests, had agreed to adhere to study requirements, and had stopped lactating at the time of screening or had temporarily ceased breastfeeding while receiving the study drug until four days after the end of infusion. Eligible participants also had to have a negative pregnancy test before initiation of study drug, had a major depressive episode with onset no earlier than the third trimester and no later than four weeks after delivery, as determined by SCID for DSM‐V Axis I Disorders, had a qualifying HAM‐D total score (≥ 26 for study 1; 20 to 25 for study 2) before infusion, were six months post partum or less at screening, were willing to delay the start of new pharmacotherapeutic drug regimens from study drug infusion until completion of 72 hour assessments, and agreed to use an approved form of birth control during the study and for 30 days thereafter. Women with onset of post‐partum depression in the peripartum period were included on the basis of previous literature demonstrating postpartum depression development in this time period. Participants taking prescribed psychotropic medication at baseline had to be at a stable dose 14 days before screening until completion of the 72 hour assessments." **Exclusion criteria**: "individuals were excluded if they had renal failure requiring dialysis, fulminant hepatic failure, anaemia (baseline haemoglobin < 10 g/dL), known allergy to allopregnanolone or to progesterone, active psychosis (determined by investigator), medical history of schizophrenia, bipolar disorder, or schizoaffective disorder, had attempted suicide during the current episode of postpartum depression, or a history of alcohol or drug abuse in the previous 12 months (by self‐report or drug screening). Individuals were also excluded if they had been exposed to another investigational medication up to 30 days before screening, had previously participated in this study or other studies of brexanolone injection or had electroconvulsive therapy up to 14 days before screening or had therapy planned within seven days after infusion." **Number recruited**: n = 108: brexanolone n = 54, placebo n = 54 **Number dropped out**: n = 8: brexanolone n = 6, placebo n = 2 **Number analysed**: n = 104: brexanolone n = 51, placebo n = 53 **Age, mean (SD) years**: brexanolone 28.4 (6.1), placebo 27.4 (5.9) **Severity of PND, mean HAM‐D (SD) score**: brexanolone 22.6 (1.6), placebo 22.7 (1.6) **Onset of PND**: third trimester: brexanolone n = 22%, placebo n = 22%; within 4 weeks of delivery: brexanolone n = 78%, placebo n = 78%; **Previous PND:** brexanolone n = 35%, placebo n = 39% **Family history of PND**: brexanolone n = 31%, placebo n = 24% **Physical health comorbidities**: not reported **Mental health comorbidities**: depression (non‐PPD): brexanolone n = 24%, placebo n = 33%; anxiety: brexanolone n = 31%, placebo n = 35%; premenstrual dysphoric disorder: brexanolone n = 4%, placebo n = 2%; other psychiatric disorder: brexanolone n = 4%, placebo n = 6% **Use of antidepressant medication at baseline:** brexanolone n = 17%, placebo n = 19%	
Interventions	**Brexanolone 90 μg/kg/h:** single continuous infusion of study drug for 60 h according to the following schedule: 30 μg/kg per h (0–4 h); 60 μg/kg per h (4–24 h); 90 μg/kg per h (24–52 h); 60 μg/kg per h (52–56 h); 30 μg/kg per h (56–60 h) **Placebo**: matching infusion **Duration**: 60 hours	
Outcomes	**Primary outcome:** change from baseline in HAM‐D total score at 60 h post‐infusion **Secondary outcomes:** mean HAM‐D total score, CGI response, change from baseline in MADRS, EPDS and HAM‐D sub‐scale, PHQ and GAD‐7 **Time points:** 0, 2, 4, 8, 12, 24, 36, 48, 60, and 72 h after infusion and follow‐up days 7 and 30	
Notes	Funded by Sage Therapeutics, Inc. We corresponded by email with Sage Therapeutics Inc. We were sent by Sage (on 28 September 2022) response and remission data for this study at day 30. We requested these data as they were only presented graphically in the published manuscript, and they were the most distal time point (Meltzer‐Brody 2018 Study 2). When we decided later to request data for the other time points, we received no response from Sage and instead used PlotDigitizer to extract data from Figures S1 and S2 of Meltzer‐Brody 2018 Study 2.	
***Risk of bias***		
**Bias**	**Authors' judgement**	**Support for judgement**
Random sequence generation (selection bias)	Low risk	Randomisation schedule provided by an independent third party
Allocation concealment (selection bias)	Low risk	Only the study pharmacist was aware of treatment allocation.
Blinding of participants and personnel (performance bias) All outcomes	Low risk	Participants and study personnel were masked to group allocation.
Blinding of outcome assessment (detection bias) All outcomes	Low risk	The study team did not have access to the trial database until the final enrolled participant had completed their final visit.
Incomplete outcome data (attrition bias) All outcomes	Low risk	Reasons for participants discontinuing the study are clearly explained. Discontinuation rates appear balanced across groups.
Selective reporting (reporting bias)	Low risk	All prespecified outcomes are reported.
Other bias	Unclear risk	The trial was funded by the drug manufacturer, who appears to have had a considerable role in the design and conduct of the trial. Many of the authors also declared financial conflicts of interest as employees or stock owners of the drug manufacturer. It is unclear if these circumstances may have biased the trial findings.

**Table:** NCT03228394

***Study characteristics***		
Methods	**Study design:** RCT **Location:** USA **Setting:** outpatient clinics **No. of centres:** not reported **Dates of study**: June 2017 to May 2019 **Total duration of study:** 24 months **Recruitment**: not reported **Randomisation method:** not reported	
Participants	**Inclusion criteria**: participant experienced a major depressive episode (diagnosed according to MINI 7.0 interview), which started between the start of the 3rd trimester and 4 weeks following delivery; given birth in the last 6 months; HAMD‐17 score of ≥ 26 at screening; must agree to stop breastfeeding from start of study treatment or must agree to temporarily cease giving breast milk to her infant(s). **Exclusion criteria**: current or past history of any psychotic illness, including major depressive episode with psychotic feature; history of suicide attempt within the past 3 years; active suicidal ideation; history of bipolar I disorder; history of seizure disorder. **Number recruited**: n = 91; ganaxolone n = 46 (ganaxolone group 1 n = 5, ganaxolone group 2 n = 15, ganaxolone group 3 n = 10, ganaxolone group 4 n = 16); placebo n = 45 (placebo group 1 n = 4, placebo group 2 n = 14, placebo group 3 n = 10, placebo group 4 n = 17) **Number dropped out**: n = 12: ganaxolone n = 5; placebo n = 7 **Age, mean (SD) years**: ganaxolone group 1: 24.4(6.27), ganaxolone group 2: 27.0 (7.11), ganaxolone group 3: 25.0 (4.11), ganaxolone group 6: 28.8 (4.97); placebo group 1: 25.3 (2.22), placebo group 2: 26.4 (6.02), placebo group 3: 26.4 (5.7), placebo group 6: 24.9 (5.1)	
Interventions	**Ganaxolone group 1:** IV infusion at rate of 4 milligram per hour (mg/h) (16 mL/h) for 48 hours, then 2 mg/h for 12 hours **Ganaxolone group 2:** IV infusion at rate of 8 mg/h for 48 hours, then 4 mg/h (8 mL/h) for 12 hours **Ganaxolone group 3:** IV bolus of 12 mg (24mL)over 2 minutes; then 12 mg/h (24 mL/h) for 48 hours and then 6 mg/h (12 mL/h) for 12 hours. **Ganaxolone group 6:** IV infusion at rate of 20 mg/h (40 mL/h) for 6 hours followed by 900 mg (4 capsules) orally at dinner for 28 days **Placebo group 1:** IV infusion at rate of 16 mL per hour (mL/h) for 48 hours, then 8 mL/h for 12 hours **Placebo group 2:** IV infusion at rate of 16 mL/h for 48 hours, then 8 mL/h for 12 hours **Placebo group 3:** IV bolus of 24 mL over 2 minutes; then 24 mL for 48 hours and then 12 mL/h for 12 hours. **Placebo group 6:** IV infusion at rate of 40 mL/h for 6 hours; then 900 mg PBO (4 capsules) orally at dinner for 28 days	
Outcomes	**Primary outcomes**: change from baseline in HAMD‐17 total score **Secondary outcomes**: response defined as ≥ 50% reduction from baseline in total HAMD‐17 score, remission defined as total HAMD‐17 score ≤ 7, change from baseline in EPDS total score, change from baseline in STAI6 total score, CGI response **Time points:** 0, 6, 12, 24, 28, 48, 60, and 72 h after infusion and follow‐up days 8, 11, 15, 22, 29, 34, 36, 57, 71	
Notes	Funded by Marinus Pharmaceuticals, Inc. We corresponded by email with Marinus Pharmaceuticals to confirm the status of this trial registered on ClinicalTrials.gov. It was included after additional data were supplied. When extracting data for this trial, we noted that results posted on ClinicalTrials.gov included the outcome severity of depression, measured with HAM‐D, at day 29 for the intravenous ganaxolone and placebo cohorts. However, we later noticed that the data for this outcome, at this time point, in these 6 treatment groups, had disappeared, with no record of the results being amended. We contacted ClinicalTrials.gov who advised us to contact Marinus Pharmaceuticals. Their response was that the change on ClinicalTrials.gov was made to enhance clarity that no participants in these treatment cohorts were analysed at this time point (the statistical analysis plan reports that HAMD‐17 was evaluated at day 29 on cohort 6 only).	
***Risk of bias***		
**Bias**	**Authors' judgement**	**Support for judgement**
Random sequence generation (selection bias)	Unclear risk	"randomised" Not enough information to permit judgement of low risk
Allocation concealment (selection bias)	Unclear risk	Not enough information to permit judgement
Blinding of participants and personnel (performance bias) All outcomes	Low risk	"double‐blind"
Blinding of outcome assessment (detection bias) All outcomes	Low risk	Outcomes assessor was masked
Incomplete outcome data (attrition bias) All outcomes	Unclear risk	Insufficient reporting of drop outs and reasons for discontinuation on Clinical Trials website.
Selective reporting (reporting bias)	Unclear risk	Not enough information to permit judgement
Other bias	Unclear risk	Not enough information to permit judgement

**BIMF**: Barkin Index of Maternal Functioning; **CGI‐I:** Clinical Global Impression: Improvement; **DSM**: Diagnostic and Statistical Manual of Mental Disorders; **ECG:** electrocardiograph; **ECT**: electroconvulsive therapy; **EPDS**: Edinburgh Postnatal Depression Scale; **GAD**: generalised anxiety disorder; **HAM‐A**: Hamilton Anxiety Rating Scale; **HAM‐D**: Hamilton Rating Scale for Depression; **HIV**: human immunodeficiency virus; **IV**: intravenous; **MADRS**: Montgomery and Åsberg Depression Rating Scale; **PHQ**: Patient Health Questionnaire; **PND**: postnatal depression; **PPD:** postpartum depression; **RCT**: randomised controlled trial; **SCID**: Structured Clinical Interview for DSM‐IV Axis I Disorders; **SD**: standard deviation; **STAI6:** Spielberger State‐Trait Anxiety Inventory 6‐item Version

### Characteristics of excluded studies [ordered by study ID]

**Table:**

Study	Reason for exclusion
Clayton 2020	Ineligible patient population: major depressive disorder
Gunduz‐Bruce 2019	Ineligible patient population: major depressive disorder
Hoffmann 2019	Ineligible patient population and study design. Healthy lactating women who received brexanolone.
NCT03460756	Not a randomised controlled trial. This appeared at first to be an RCT. When we checked the study results published on ClinicalTrials.gov, we noticed there were no placebo data. We contacted Marinus Pharmaceuticals, who confirmed in correspondence that "after completing 4 open‐label cohorts (those published), the study was stopped and double‐blind, placebo‐controlled trials were never started. However, the title had never been updated to reflect this change".
NCT03864614	Ineligible patient population: major depressive disorder
NCT03924492	Not a randomised controlled trial
NCT04273191	Phase 4 study withdrawn (Sage Therapeutics decided not to proceed with this study)
NCT04442490	Ineligible patient population: major depressive disorder
Riesenberg 2022	Open‐label study of brexanolone ‐ no comparator

### Characteristics of studies awaiting classification [ordered by study ID]

**Table:** Reisdorf 2021

Methods	"Phase III study"
Participants	"153 young mothers"
Interventions	Participants treated with "zuranolone versus placebo for 14 days"
Outcomes	"Compared with placebo, administration of zuranolone tablets resulted in significantly greater improvement in symptoms according to the Hamilton Depression Scale on day 15. Patients in the zuranolone group also performed better on secondary endpoints."
Notes	All data extracted from the study abstract, published in German, using Google translate, and copied verbatim here. Abstract available at: https://www.krankenhauspharmazie.de/heftarchiv/2021/11/zuranolon‐bessert‐ham‐d‐17‐score‐waehrend‐und‐nach‐2‐woechiger‐therapie‐1.html

## Differences between protocol and review

We adjusted the title of the review to 'Brexanolone, zuranolone and related neurosteroid GABA_A_ receptor positive allosteric modulators for postnatal depression' (from 'Brexanolone and related neurosteroid GABA_A_ positive allosteric modulators for postnatal depression' (Wilson 2021)).

In our protocol (Wilson 2021), we specified that three or more studies would be required for meta‐analysis. However, after consulting with the Cochrane Editorial team, we decided to meta‐analyse the zuranolone studies (of which there were only two), based on the clinical homogeneity of these studies.

In our protocol, we stated the following in the 'Measures of treatment effect' section: "If a meta‐analysis could be conducted for continuous data, we analysed these by calculating the mean difference (MD) between groups, if studies used the same outcome measure for comparison. If studies used different outcome measures to assess the same outcome, we calculated standardised mean difference (SMD) and 95% CIs. Where trial arm‐level data were unavailable, we used mean differences and their SE in meta‐analyses using the generic inverse‐variance method."

However, after seeing the format of the data where between‐group mean differences (MDs) were presented in the trials, we were advised by the Cochrane Methods Support Unit to use the generic inverse‐variance method for our depression severity analyses. The mean differences are adjusted for various factors since they are derived from a mixed‐effects model. It was preferable to use these as they take into account both between‐ and within‐participant variation in the outcome measurements, which traditional MDs fail to do.
